# Aroma Identification and Traceability of the Core Sub-Producing Area in the Helan Mountain Eastern Foothills Using Two-Dimensional Gas Chromatography and Time-of-Flight Mass Spectrometry and Chemometrics

**DOI:** 10.3390/foods13223644

**Published:** 2024-11-15

**Authors:** Yuanke Zhang, Zefang Cui, Jianing Li, Mengyuan Wei, Yue Wang, Wenguang Jiang, Yulin Fang, Xiangyu Sun, Qian Ge

**Affiliations:** 1 College of Enology, Northwest A&F University, Yangling 712100, China; zhangyk2001@nwafu.edu.cn (Y.Z.); ccczf@nwafu.edu.cn (Z.C.); lijianing0357@nwafu.edu.cn (J.L.); weimengyuan@nwafu.edu.cn (M.W.); wangyue11@nwafu.edu.cn (Y.W.); cyjiangwenguang@163.com (W.J.); fangyulin@nwsuaf.edu.cn (Y.F.); 2 Ningxia Institute of Agricultural Products Quality Standards and Testing Technology, Yinchuan 750002, China

**Keywords:** eastern foothills of Helan Mountain in Ningxia, wine, GC×GC-TOFMS, aroma, producing area traceability

## Abstract

The combination of volatile compounds endows wines with unique aromatic characteristics and is closely related to their geographical origins. In the pursuit of origin identification and the subdivision of homogeneous production areas, clarifying the characteristics of production areas is of great significance for improving wine quality and commercial value. In this study, GC×GC-TOFMS technology was used to analyze the aroma characteristics of “Cabernet Sauvignon” wines from 26 wineries in the Helan (HL), Yinchuan (YC), Yongning (YN), Qingtongxia (QTX), and Hongsibu (HSP) sub-producing areas in the eastern foothills of Helan Mountain in Ningxia, China. The results indicate a gradual increase in relative humidity from the southern part of Ningxia, with the YN sub-region showing optimal fruit development and the QTX region having the highest maturity. A total of 184 volatile compounds were identified, with 36 compounds with an OAV > 1, crucial for the aroma profiles of primarily fermentation-derived alcohols and esters. An aromatic vector analysis revealed that “floral” and “fruity” notes are the primary characteristics of Cabernet Sauvignon wines from the Helan Mountain East region, with lower maturity aiding in the retention of these aromas. By constructing a reliable OPLS-DA model, it was determined that 15 substances (VIP > 1) played a crucial role in identifying production areas, among which phenylethyl alcohol and isoamyl alcohol were the main contributors. In addition, a Pearson correlation analysis showed a negative correlation between sunlight duration during the growing season and benzyl alcohol accumulation, while a significant positive correlation was observed during the ripening period. Due to the critical role of phenyl ethanol in identifying producing areas, this further demonstrates that sunshine conditions may be a key factor contributing to the differences in wine flavor across regions. This study offers a theoretical foundation for understanding the relationship between climatic factors and flavor characteristics, addressing the issue of wine homogenization in small production areas, clarifying typical style characteristics, and establishing a traceability technology system based on characteristic aroma.

## 1. Introduction

Over the past few decades, the global wine industry has witnessed significant growth. A previous study indicates that consumers exhibit a growing preference for wines characterized by unique sensory profiles and are willing to pay a premium price for these products [1]. This trend has aroused increased attention to concepts such as Terroir, regional characteristics, and geographical typicality [2]. The concept of “Terroir” delineates the relationship between the sensory characteristics of wine and its geographical origin, encompassing geographical location, soil type, topography, and climatic conditions, as well as grape cultivation and winemaking practices [3]. In this case, some studies have achieved geographical typicality identification by measuring the mineral elements [4], metabolomics [5], stable isotopes [6], and soil characteristics of vineyard soils [7]. With the introduction of the concept of “microbial soil” [8], it has become a tendency to subdivide smaller or more homogeneous areas. For example, the Claire Valley is divided into five sub-regions [9]. Some producers evenbegan to imitate the French village classification system based on the township of the production area segmentation [10].

Climatic conditions exert a significant influence on the yield and quality of grapes and wines. Appropriate solar radiation and temperature can facilitate the synthesis of phenolic compounds and volatile compounds [11]. The air temperature at maturity has a decisive influence on the chemical composition, aroma, and color of grape berries, thereby directly affecting the sensory characteristics and overall quality of the final wine [12]. Different combinations of volatile compounds give wines a variety of aromatic characteristics. Zhang et al. demonstrated that wines from different regions display unique chemical characteristics, and the influence of region on wine diversity even exceeds the variety difference [13]. In particular, geographical typologies are often closely related to aroma characteristics [14], which underscores the importance of identifying volatile compounds associated with the geographical locations of vineyards. In homogenized producing areas, even small differences in geographical characteristics can result in significant variations in wine aroma. However, traditional methods are often incapable of accurately identifying and differentiating these volatile compounds that are closely associated with geographical location. As a promising flavor analysis approach, comprehensive two-dimensional gas chromatography and time-of-flight mass spectrometry (GC×GC-TOFMS) outperforms traditional gas chromatography–mass spectrometry (GC-MS) in terms of sensitivity, precision, and separation capability [15]. At present, GC×GC-TOFMS has been widely used in food and beverage flavor research, including tea [13], liquor [16], beer [17], wine [18], etc., and achieved good results. Using the data collected by GC×GC-TOFMS for non-targeted analysis, combined with supervised or unsupervised experimental design, samples can be effectively characterized and distinguished, and key components can be identified [19].

The eastern foothills of the Helan Mountains in Ningxia are situated at the intersection of the alluvial slope plain of the Helan Mountains and the alluvial plain of the Yellow River [20]. The area has a typical temperate continental climate and is one of the important wine-producing areas in China. At present, the production area has multiple core sub-producing areas such as Shizuishan, Helan, Yinchuan, Yongning, Qingtongxia, and Hongsibu. Although the eastern foothills of Helan Mountain production area has been certified as a national geographical origin, issues still remain in the wines from this region, including indistinct typical aroma characteristics and product homogenization. Moreover, there is a risk of counterfeiting geographical labels and inferior quality products. At present, most studies on the traceability of wine-producing areas in China focus on the identification [21,22] of large producing areas, such as provincial classification (such as in Ningxia and Xinjiang [13]), and the research on subdivided producing areas is relatively insufficient.

In this study, a unified brewing process was employed to exclude the influence of the winemaker style. Then, GC×GC-TOFMS technology was utilized to analyze the aroma characteristics of wines from five core sub-producing areas at the eastern foothills of Helan Mountain in Ningxia, and OPLS-DA technology was applied to identify the producing areas. Furthermore, the relationship between climatic conditions and flavor characteristics of the producing areas was explored using a Pearson correlation analysis. This study aims to reveal the relationship between climatic factors and flavor characteristics and provide a theoretical foundation for addressing the homogenization of wine in small producing areas, clarifying typical style characteristics and establishing a traceability technology system based on characteristic aroma. Furthermore, this research method can offer a reference for the segmentation of other regions, facilitating a profound understanding of the diverse flavors of producing regions, supporting precise cultivation and brewing management, enhancing the quality of product style, and promoting the sustainable development of the global wine industry.

## 2. Materials and Methods

### 2.1. Chemicals

NaCl (≥99%), methanol (≥99%), ethanol, glucose, tartaric acid, and NaOH (≥97%) were provided by Yangling Chemical Plant (Shaanxi, China). 4-Methyl-2-pentanol (purity ≥99%), all compounds mentioned in Table 1, and n-alkanes (C_7_–C_40_) were purchased from Sigma-Aldrich (St. Louis, MO, USA). Purified water was obtained from the Milli-Q purification system (Milli-Q Advantage A10, Millipore, Bedford, MA, USA).

### 2.2. Grape Materials and Winemaking

In September 2023, the authors collected 26 samples of “Cabernet Sauvignon” (*Vitis vinifera* L.cv. “Cabernet Sauvignon”) from 26 wineries located in the five core sub-producing areas of Helan (HL), Yinchuan (YC), Yongning (YN), Qingtongxia (QTX), and Hongsibu (HSP) in the eastern foothills of Helan Mountain in Ningxia. The geographical coordinates of the sample collection points range from 105°54′07′′ E to 106°17′80′′ E and from 37°30′52′′ N to 39°71′35′′ N, with an altitude ranging from 1142.6 m to 1512.7 m above sea level. The specific geographical location information is shown in Table 2.

During the harvest period, small-scale fermentation experiments were conducted. The specific brewing process is as follows: 20 kg of grapes was randomly picked, and the stems were broken. Three portions were put into a 20 L fermenter, and 60 mg/L of SO_2_ and 25 mg/L of pectinase (Lallzyme Ex, Lallemand, Lyon, France) were added. After 24 h, 200 mg/L of fully activated commercial Lalvin strain D254 yeast (Laffort, Bordeaux, France) was added. During the fermentation, the cap was pressed three times a day, and the temperature and specific gravity were monitored. After the end of alcohol fermentation, the residue was separated, clarified, and bottled. The specific physical and chemical indicators of the wine are shown in Table 3.

### 2.3. Meteorological Data Collection

The phenological period of wine grapes in Ningxia can be divided into four stages: the germination period (April), flowering period (May and June), coloring period (July and August), and mature period (September). To distinguish the effects of climatic factors at each growth stage, this study analyzed the monthly average temperature, relative humidity, rainfall, and sunshine duration of grape growth and development stages in each sub-producing area in 2023. Meteorological data are provided by WheatA (https://www.wheata.cn, accessed on 1 August 2024). The data of each production area are shown as the mean values of the meteorological data of the three meteorological stations closest to the sampling points, and the specific coordinates of the meteorological stations involved are shown in Table 4.

### 2.4. Analysis of Physical and Chemical Parameters of Grape and Wine

All parameters were tested in triplicate. The determination of the 100-grain weight was based on the method of Zhang et al. [23]. Specifically, 100 fruit grains were randomly selected and weighed, and the average mass (g) was calculated. Total Soluble Solid (TSS) was measured using a PAL-1 digital Abbe refractometer (PAL-1; Atago Co. Ltd., Tokyo, Japan). The results were expressed as °Brix. The pH value was measured using a PHS-3E pH meter (PB-10; Sartorius, Göttingen, Germany). The determination of reducing sugar (RS), Titratable Acid (TA), and alcohol contents was based on the standard method of the International Grape and Wine Organization (OIV, 2017). The TSS/TA Ratio was calculated as the ratio of the Total Soluble Solid content to the Titratable Acid content.

### 2.5. GC×GC-Q-TOF MS Analysis

All samples were analyzed by GC×GC-TOFMS (comprehensive two-dimensional gas chromatography quadrupole time-of-flight mass spectrometry), which was equipped with an Agilent 8890 GC/7250 gas chromatography tandem quadrupole time-of-flight mass spectrometer (Agilent, Santa Clara, CA, USA), a headspace solid-phase microextraction automatic sampler (GERSTEL, Mülheim an der Ruhr, Germany), and a solid-state thermal modulator (J&X Technologies, Shanghai, China). Amounts of 5 mL of wine sample, 1 g of Nacl, and 10 μL of 4-methyl-2-pentanol (324.4 μg/L, internal standard) were placed in a 20 mL headspace vial, immediately sealed with a screw cap, and placed in an automatic sampler tray. The solid-phase microextraction needle fiber was made of divinylbenzene/carbon molecular sieve/polydimethylsiloxane (DVB/C–WR/PDMS; fiber thickness: 50/30 μm; Thermo Fisher Scientific, Carlsbad, CA, USA). SPME fibers were extracted at 45 °C at 250 rpm for 30 min and then quickly inserted into the syringe port at 250 °C for thermal desorption (3 min).

GC×GC consisted of a DB-WAX column (30 m × 0.25 mm × 0.25 μm, Agilent, Santa Clara, CA, USA) and a DB-17MS column (1.85 m × 0.180 mm × 0.18 μm, Agilent, Santa Clara, CA, USA). The initial temperature of the column box was 50 °C, which was increased to 230 °C at a rate of 3 °C/min and maintained for 2 min. Splitless injection was carried out. The carrier gas was helium (99.999%), and the constant flow rate was 1 mL/min. The ion source temperature was maintained at 230 °C, and the EI ionization energy was 70 eV. The mass scan range was set to 45–500 m/z at full scan mode, the mass spectrometry sampling rate was 50 times/s, and the solvent delay was 2 min. For the heating and cooling stages, the solid-state modulator used the SV modulation column (C_6_~C_40_), the modulation period was 4 s, and the cold zone temperature was maintained at −50 °C.

### 2.6. Qualitative and Quantitative Analyses

Canvas Panel 2.5.710 software (J&X Technologies, Shanghai, China) was used to process the original data. The signal-to-noise ratio (S/N) was 10 for data processing, and substances with positive and negative matching degrees greater than 700 were screened. N-alkanes (C_7_~C_40_) were used to calculate RI. The RI and NIST 20 databases were used to identify compounds.

The quantification of volatile compounds is achieved by constructing a standard curve (Table 1). The simulated wine solution (13%vol alcohol, 3 g/L glucose, and 6 g/L tartaric acid, pH 3.5) was prepared according to the study of Ling et al. [24]. The mixed standard solution was diluted with methanol in a 2^n^ gradient to obtain nine distinct concentrations. Subsequently, these diluted solutions were combined with the internal standard substances (4-methyl-2-pentanol, 324.4 μg/L) and added to the simulated wine solution for further analysis. The corresponding standard curves were drawn according to the peak area ratio and concentration ratio of standard compounds and internal standard substances, and the determination coefficient (R^2^) was calculated. Each gradient was repeated three times. Under the same analytical conditions, the content of volatile compounds in the sample was calculated according to the peak area of the sample to be tested and the corresponding linear equation of the standard. For compounds without a standard curve, the standard curve of a compound with a similar material type and a similar number of carbon atoms is used for quantification [25].

### 2.7. Odor Active Value and Aroma Series

Based on the quantitative results and the corresponding odor thresholds, the odor activity value (OAV) of the volatile compounds was calculated to illustrate their sensory contribution to the characteristic aroma of the wine. The OAV is calculated by the equation OAV = c/t, where c is the concentration of volatile components, and t represents the odor threshold of the compound (Table 2).

### 2.8. Statistical Analysis

All data were analyzed using SPSS (version 22.0; IBM, Armonk, NY, USA) for a one-way analysis of variance (ANOVA) and Tukey’s test (*p* < 0.05). Orthogonal partial least squares discriminant analysis (OPLS-DA) and plotting were performed using SIMCA-P 14.1 (Umetrics, Umeå, Sweden). Images were drawn by Origin 2021 (OriginLab Corporation, Northampton, MA, USA) and ChiPlot (https://www.chiplot.online/, accessed on 28 August 2024). All chemical variables were normalized before multivariate statistical analysis.

## 3. Results

### 3.1. Weather Data Analysis of Each Sub-Producing Area

As shown in Table 5, significant differences exist in the four phenological stages within each producing area. Throughout the entire phenological period of grape growth, the monthly average temperature ranged from 9.57 °C to 26.04 °C, the relative humidity ranged from 36.13% to 53.94%, and the average precipitation ranged from 11.02 mm to 43.40 mm. Furthermore, the monthly total sunshine hours in each region range from 152.27 h to 215.71 h. In the Ningxia Plain, the altitude gradually decreases from south to north and from west to east, which leads to generally higher temperatures in the northern producing areas compared with the southern ones [26]. In all stages, the monthly average temperature in the YN region is generally the highest, whereas that in the Hongsibu production area is the lowest. In the wine grape reserve at the eastern foot of Helan Mountain, the Hongsibu area is designated as a cool climate zone, whereas most of the other areas fall into a moderate climate zone. The study conducted by Tan et al. [27] demonstrated that the relative humidity of the grape growing area in Ningxia progressively increased with the geographical location towards the south, and this study also corroborates this finding.

The HSP region is regarded as the coldest wine-producing area in Ningxia, with the highest relative humidity and precipitation at each stage and the lowest monthly average temperature. Unexpectedly, the HSP region had the most sunshine hours among the five production areas before maturity, whereas the HL production area had the most sunshine hours at maturity, which amounted to 157.65 h. In all producing areas, the average temperature and precipitation continued to increase during the fruit development stage and decreased after reaching their highest values during the berry-turning period. Meanwhile, the relative humidity gradually increased during the grape-ripening process, which might affect the flavor and maturity of grapes.

### 3.2. Basic Physical and Chemical Properties of Grapes and Wine

**Figure 1:**
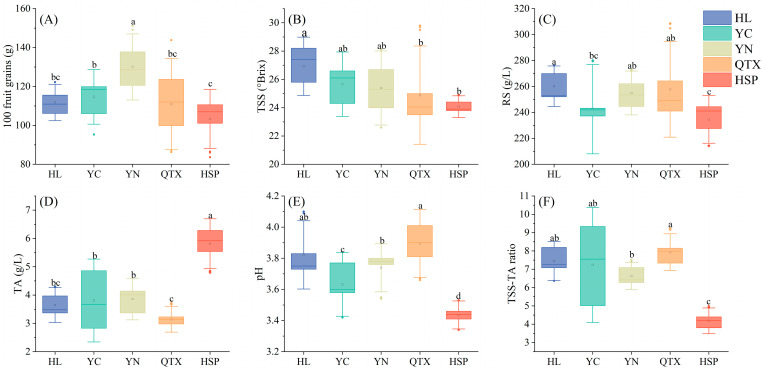
Basic physical and chemical parameters of wine grapes in each producing area: (**A**) 100-grain weight (g); (**B**) Total Soluble Solids (°Brix); (**C**) reducing sugar (g/L); (**D**) Titratable Acid (g/L); (**E**) pH value; (**F**) TSS-TA ratio. Different letters indicate that there are significant differences between different producing areas (*p* < 0.05).

To better focus on the differences among producing areas, the basic physical and chemical indexes of wine grapes and wines in each producing area were averaged, and the results are presented in Figure 1.

The 100-grain weight is a crucial index for fruit growth and yield prediction. Among them, the 100-grain weight of grapes in the YN region is the highest (130.06 g), indicating that the grape clusters in this producing area are large and well developed. In contrast, the 100-grain weight of fruits in the QTX region varies considerably, and fruit development is uneven. The contents of reducing sugar and TSS were consistent in each producing area. The contents of reducing sugar (260.19 g/L) and TSS (26.92 °Brix) in the HL region were significantly higher than those in other producing areas (*p* < 0.05). It is speculated that this is related to the higher soil fertility in this area, which facilitates the absorption of nutrients and the accumulation of sugar in grapes [28]. The reducing sugar content, TSS, and pH values of the HSP region were the lowest, while the Titratable Acid content (5.81 g/L) was significantly the highest (*p* < 0.05), which was closely associated with the lower average temperature and higher precipitation in the producing area. The standard deviation of the reducing sugar content in the QTX and YN producing areas was the largest, indicating that there was a significant difference in the fruit sugar content between these two producing areas. The TSS-TA ratio is an important index for measuring the maturity of grapes [29]. The average TSS-TA ratio in most producing areas is above 6.63. Among them, the TSS-TA ratio (7.94) in the QTX region is the highest, indicating that the fruit maturity in this producing area is the best.

The concentration of alcohol in wine mainly depends on the sugar content during grape harvest [30]. The basic physicochemical properties of wine are shown in Table 3. This study demonstrates that the alcohol content in wine was consistent with the reducing sugar content in grapes. The alcohol content (13.27%vol) and residual sugar content (3.37 g/L) in the HL region were the highest, and they were significantly higher than those in other producing areas (*p* < 0.05). In general, there was no difference in the alcohol content among different producing areas (*p* > 0.05). Titratable Acid and pH can directly influence the overall sensory characteristics of wine. A higher pH will result in “softer” wine in the mouth, with higher perceived viscosity and lower perceived acidity [31]. The pH value of the QTX region was 3.59, which was significantly higher than that of other producing areas (*p* < 0.05). The highest Titratable Acid content (5.60 g/L) was found in the HSP region, while there was no significant difference among the HL, YC, and YN producing areas, and the lowest content (5.03 g/L) was found in the QTX region.

### 3.3. Analysis of Volatile Compounds

**Figure 2:**
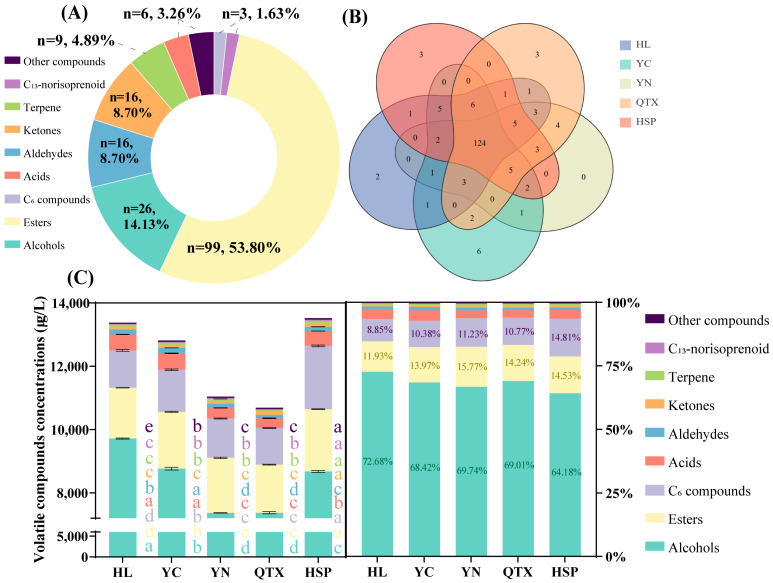
An analysis of the volatile compounds in wines from different producing areas. (**A**) The proportion of 9 volatile compounds. (**B**) A Wayne diagram of volatile compounds; (**C**) The contents and percentages of volatile compounds. Different letters indicate significant differences (*p* < 0.05) between different producing areas.

According to the results of the quantitative analysis, the content of total volatile compounds in the five sub-producing areas ranged from 10,687.19 μg/L to 13,520.80 μg/L, and a total of 184 volatile compounds were identified (as shown in Table S1). As presented in Figure 2A, these compounds are divided into nine categories, of which esters are the most abundant, accounting for 53.80% (99 species), followed by alcohols, accounting for 14.13% (26 species), and 16 aldehydes and ketones (8.70%), nine terpenes, six acids, three C_6_ compounds and C_13_-norisoprenoids, and six other compounds. As depicted in Figure 2B, it is evident that there are differences in the number of common volatile compounds among the five producing areas. The non-overlapping regions represent compounds unique to each producing area. A total of 124 volatile compounds were detected in all of the producing areas, while some specific compounds were detected only in specific producing areas. For instance, in the HL producing area, compounds such as methyl cinnamate and butyl hexanoate were detected. Similarly, in the YC producing area, n-butyl acrylate, 2-methoxy-3-isopropyl pyrazine, 2-phenylmethyl hexanoate, 2,4,7,9-tetramethyl-5-decyne-4,7-diol, nerol acetate, and Edulan I were detected (Table S1).

Figure 2C shows the total content and proportion of various volatile compounds in the five producing areas. It can be observed from the figure that the proportion of various compounds was consistent among the five producing areas. Among them, fermentation-derived alcohols and esters represent the primary volatile compounds found in wine, corroborating previous findings [13], accounting for more than 64.18% and 11.93%, respectively. In this study, only three C_6_ compounds were identified, namely n-hexanol, cis-2-hexen-1-ol, and trans-3-hexen-1-ol, which were defined as the sources of green, herbaceous, and vegetable aromas [3]. The wine produced in HL had the highest alcohol content, and its main volatile components included phenylethyl alcohol (5531.87 μg/L), isoamyl alcohol (3369.02 μg/L), and n-hexanol (1904.29 μg/L). Among them, isoamyl alcohol and n-hexanol had irritating and spicy odor characteristics [32], which are related to the odors of herbaceous plants. When the concentration was lower than 300 mg/L, it was conducive to improving the complexity of the wine. Phenylethyl alcohol, which is mainly formed by yeast metabolism, is a key odorant contributing to honey and flower aromas [33].

Ester compounds are mainly composed of acetate and fatty acid ethyl esters, such as isoamyl acetate, ethyl caprylate, ethyl caprate, and ethyl caproate. In this study, isoamyl acetate and ethyl caprylate were the most abundant esters in all wines, with concentrations of 221.50 μg/L–365.31 μg/L (HSP > YN > YC > QTX > HL) and 116.03 μg/L–196.33 μg/L (HSP > YC > YN > HL > QTX), respectively, and they played a key role in the formation of fruit and banana flavors. An appropriate fatty acid content can balance the aroma of esters in wine [34]. When the concentration of fatty acids is in the range of 4 mg/L–10 mg/L, it can impart a mild and pleasant aroma to the wine, and when the concentration exceeds 20 mg/L, it may have a negative impact on the wine’s aroma [25]. The three straight-chain fatty acids (caproic acid, octanoic acid, and decanoic acid) identified in this study were significantly different between different producing areas. Specifically, the highest caproic acid content was found in the YC region, followed by the HL region, while these two fatty acids were not detected in the QTX and YN producing areas. A cool and humid environment usually leads to an increase in the content of C_6_ aldehydes in grapes, but these C_6_ aldehydes are often converted to C_6_ alcohols or acids during fermentation [35]. Therefore, the average relative humidity and precipitation in the producing area have a significant influence on the formation of fatty acids in wine. As the HSP producing area had the highest average relative humidity and precipitation, its caprylic acid and capric acid contents were significantly higher than those of other producing areas.

Aldehydes and ketones are derived from fermentation and oxidation processes, and their sensory thresholds are 100 to 10,000 times lower than the corresponding alcohols [36]. Therefore, even slight oxidation changes can have a significant impact on the aroma of wine. Phenylacetaldehyde is a Strecker degradation product, which has been identified in several types of wines and is associated with a “honey” aroma and premature oxidation [37]. The content of phenylacetaldehyde in the QTX and YN producing areas was significantly higher than that in other producing areas, whereas the content in the HSP producing area was the lowest. Among ketones, diisobutyl ketone has the highest content, with a light sweet mint flavor, and its content is significantly different among the producing areas; its content in the HSP producing area is significantly higher than that of other producing areas.

Terpenoids and norisoprene, which mainly produce floral and fruity aromas, are considered to play a crucial role in determining the typicality of wine varieties [38]. The content of monoterpenes was positively correlated with the increase in sunshine duration [13]. Because the HSP producing area had the longest sunshine duration, the monoterpene content was the highest in this area. However, the monoterpene content in the YC producing area with the shortest sunshine duration was not the lowest, which may be related to the effective accumulated temperature. Similarly, C_13_-norisoprenoid compounds (such as *β*-damascenone and *β*-ionone with apple, rose, and honey aromas) also showed similar results. These results suggest that the aroma characteristics of wines are influenced by multiple environmental factors, revealing the influence of different producing areas on the diversity and complexity of aroma compounds. C_13_-Norisoprenoid components primarily originate from the degradation of carotenoids [39], while chemical transformations occurring in wine can also contribute to the elevation of C_13_-norisoprenoids [40]. Climate conditions, especially prolonged light exposure, can markedly inhibit the synthesis of carotenoid precursor materials [41]. This is likely due to the fact that excessive light can cause photooxidative stress in plants, leading to the degradation or inactivation of enzymes involved in the biosynthesis pathway of carotenoid precursors. However, in practical production settings, extending light duration is correlated with an increase in C_13_-norisoprenoids [42]. Notably, this effect is particularly prominent when the levels of *β*-ionone and *β*-cyclocitral are elevated, which highlights the complex interplay between light conditions and the biosynthesis of carotenoid derivatives in winemaking (Figure S1).

### 3.4. Analysis of Aroma Activity (OAV)

**Figure 3:**
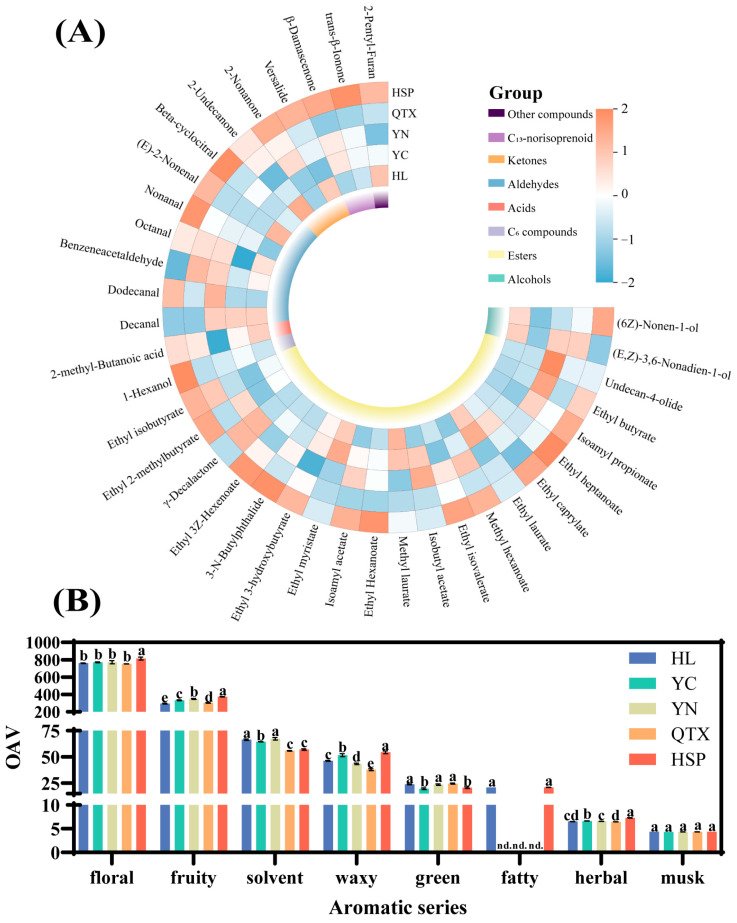
Analysis of volatile compounds based on OAV > 1. (**A**) Heat map. (**B**) Intensity of different aroma categories. Detailed explanations of aroma category attributes can be found in Table 6. Different letters indicate that there are significant differences between different producing areas (*p* < 0.05).

Odor characteristics play a key role in evaluating the flavor and quality of wine. It is generally accepted that only compounds with an odor activity value (OAV) higher than 1 can significantly influence the aroma characteristics of wine [37]. In this study, a total of 36 compounds with an OAV > 1 were identified (Figure 3). Among them, esters still accounted for the largest proportion of compounds, with a total of 19. Ethyl octanoate had the highest OAV (more than 23 in each producing area) among the esters, which contributed more to the waxy aroma. In particular, *β*-damascenone, *β*-ionone, and dodecanal were the key compounds with relatively high OAVs (mean 23.21–813.99), which were considered to be related to the aroma properties of flowers, fruits, and plants (Table 6). Generally, the OAV of aroma compounds in the HSP producing area are relatively higher, resulting in more pronounced aroma characteristics compared to other regions (Figure 3A). This characteristic is primarily manifested in the abundance of ester compounds and C_13_-norisoprene derivatives. Production practices have shown that increased light exposure can significantly enhance the levels of C_13_-norisoprene derivatives [43] and promote ester accumulation [44] in both grape berries and wine. Furthermore, while the number of compounds with an OAV > 1 in the QTX and YC producing areas is relatively similar, the overall contents of aromatic substances differ markedly. This discrepancy is likely influenced by factors such as the vineyard microclimate and other environmental variables [45].

An aroma vector is defined as “a sensory unit composed of one or more molecules with similar aroma descriptors, which are responsible for the specific sensory characteristics of a product” [36]. By constructing an aroma vector, compounds with similar odor characteristics can be classified into an aroma series, which is determined by the sum of the OAVs of each compound [45]. In this research, six aromatic series were established, including floral, fruity, waxy, green, fatty, herbal, musk, and solvent fragrances (Table 6). As shown in Figure 3B and Table 6, “floral” and “fruity” are the most important aromatic series in wine, primarily due to the low odor thresholds of *β*-damascenone and *β*-ionone [70]. Studies have shown that the maturity of grapes has a significant impact on the fruity and floral intensities of wines [71]. Specifically, the fruit maturity of the HSP producing area was low (low TSS-TA ratio), and the aroma-active components showed the highest intensities of “flower” and “fruit” aromas. Moreover, studies have shown that wines produced from grapes harvested at the same time but with lower ripeness compared to those meeting maturity standards exhibit elevated levels of *β*-damascenone and *β*-ionone [72]. This finding suggests that harvesting grapes at a relatively lower maturity can enhance the retention of these desirable aromatic characteristics, further emphasizing the importance of ripeness in the overall aromatic profile of wine. This implies that lower maturity may contribute to the preservation of these aromatic features. The aroma of the “green” series was most prominent in the YN and QTX producing areas, which was attributed to the high accumulation of C_9_ alcohol and phenylacetaldehyde. In particular, the intensity of the “fatty” series in the HL and HSP producing areas was the highest, with no significant difference between them, while the intensity of the “fatty” series in the YN, YC, and QTX producing areas was 0. There was no significant difference in the “musk” series among the five producing areas (*p* > 0.05). These results indicate that different producing areas had significant effects on the aroma characteristics of wine, underscoring the crucial role of terroir in shaping these qualities, which further emphasizes the important role of producing areas in shaping the aroma characteristics of wine. Furthermore, these results enhance our understanding of how regional influences mold wine quality and suggest opportunities for customized production practices.

### 3.5. Multivariate Statistical Analysis

**Figure 4:**
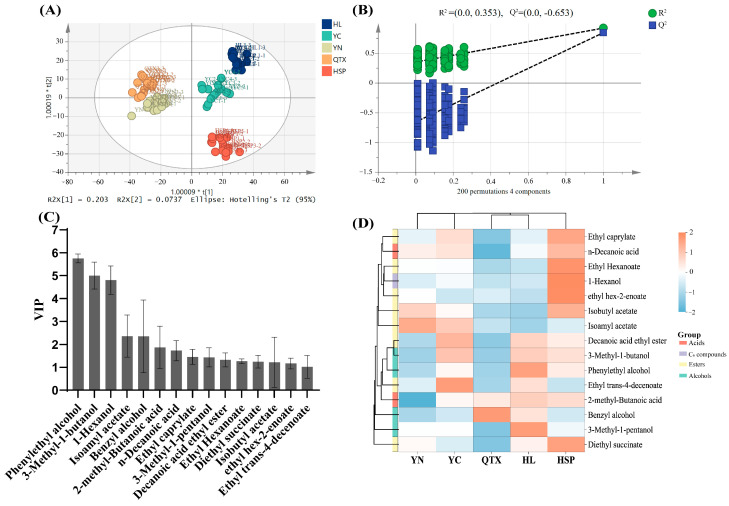
Multivariate statistical analysis of volatile compounds obtained by GC×GC-TOFMS. (**A**) OPLS-DA score plot. (**B**) Cross-validation plot through 200 permutation tests. (**C**) Substance with VIP > 1. (**D**) Heat map based on VIP > 1.

An orthogonal partial least squares discriminant analysis (OPLS-DA) is an extension of Partial Least Squares Regression (PLS-R). This method can effectively reduce the model’s complexity and enhance its interpretability without sacrificing its predictive ability [73]. In this study, five producing areas were identified by an OPLS-DA analysis, and a reliable model was constructed (*R*^2^*X* = 0.992, *R*^2^*Y* = 0.878, and *Q*^2^ = 0.776) (Figure 4A). The stability of the model was verified through 200 permutation tests. The results show that the intercepts of R^2^ and Q^2^ are 0.353 and −0.653, respectively (Figure 4B).

The results indicate that there was a clear separation trend among the producing areas. The HL and YC producing areas are located in the first quadrant, which could be attributed to the similar aroma characteristics of the wines from the geographically close planting areas [34]. YN and QTX are distributed in the second and third quadrants and are adjacent to each other. It is speculated that they are related to the similar climatic conditions in the two regions, while the HSP producing area became a clearly separated cluster. The same trend was also observed in the cluster heat map (Figure 4D). Compounds with VIP values greater than 1 are generally regarded as having a high contribution to the classification [74]. In this study, a total of 15 substances were found to have VIP values greater than 1 (Figure 4C). Although esters account for a significant proportion in terms of quantity, including ethyl octanoate, isobutyl acetate, ethyl decanoate, diethyl succinate, ethyl hexanoate, isoamyl acetate, and ethyl enoate, alcohols remain the main contributors (Figure 4C). In particular, the VIP values of phenylethanol and isoamyl alcohol were markedly higher than those of the other components, thereby further highlighting their dominant role in aroma characteristics. Previous studies have confirmed that these two compounds are important volatiles that affect the bouquet of wines and are key factors in the subtle flavor differences among wines, endowing wines with rose and whiskey flavors [75]. Although previous studies have confirmed that norisoprenoids and terpenes have an important impact on the geographical differences and typicality of wine [2], this study did not find these substances to have key roles in the model. By constructing a heat map, the differences in these substances in different producing areas can be visually presented (Figure 4D). For example, the concentration of benzyl alcohol in the QTX producing area was significantly higher than that in the other production areas; 3-methylpentanol and phenylethyl alcohol had the highest concentrations in the HL producing area, while the YN producing area had the highest concentration of isobutyl acetate and the lowest concentration of 2-methylbutyric acid. These results reflect the significant differences in the accumulation of specific compounds in each producing area and reveal the similarities and differences in chemical characteristics between regions.

### 3.6. Correlation Analysis

**Figure 5:**
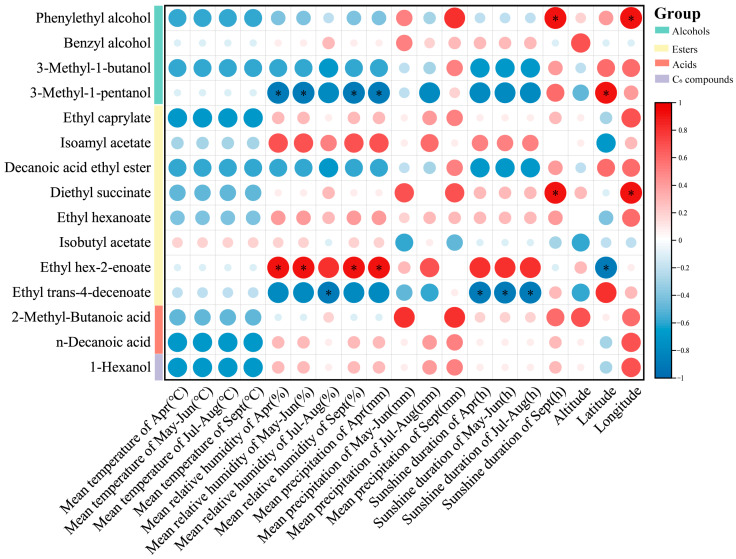
A correlation heat map between the substances of key compounds (VIP > 1) and meteorological factors. * is shown when the correlation is significant at *p* < 0.05.

The Pearson correlation coefficient method was used to analyze the correlation between key compounds (VIP > 1) and meteorological parameters in the identification of five producing areas (Figure 5).

Temperature has a significant impact on the synthesis and degradation of volatile compounds in grapes. While high temperatures generally accelerate the decomposition of these compounds, this study did not detect a significant negative correlation. Relative humidity during the phenophase almost had a significant positive correlation with the accumulation of ethyl hexadec-2-enoate but showed a significant negative correlation with the accumulation of 3-methylpentanol. Additionally, relative humidity was negatively correlated with ethyl trans-4-decenoate and manifested a significant negative correlation during the coloring period from July to August. Precipitation in April also had a significant impact on these two compounds. Specifically, there was a significant positive correlation with the accumulation of ethyl hexadec-2-enoate and a significant negative correlation with 3-methylpentanol. However, latitude had the opposite impact on the accumulation of these two compounds. Diethyl succinate has been proven to be a characteristic volatile compound of malolactic fermentation in young wine [76]. In this study, a significant positive correlation was observed between sunshine duration and the accumulation of diethyl succinate, particularly during the mature stage. Meanwhile, prior to the maturity stage, there was a significant negative correlation between sunshine duration and trans-4-decenyl acetate. After the maturity stage, this relationship transformed into a non-significant positive correlation. This indicates that the impacts of sunshine duration on different metabolites vary temporally, especially at different stages of the growth cycle. In addition, a significant positive correlation also existed between longitude and the accumulation of diethyl succinate. Phenylethyl alcohol is a rose-flavored aromatic fuel, which is a key aroma component of many wines [77]. The sunshine duration during the growth period was negatively correlated with the accumulation of phenylethyl alcohol, while during the maturity period, it was significantly positively correlated. This shift implies that climatic conditions might exert an influence on the final flavor during different growth stages by modulating the synthetic pathways of volatile compounds. Although less rainfall is usually conducive to the formation of fatty acids [13], this study found that the accumulation of sunflower acid was positively correlated with precipitation. These results reveal the complex effects of meteorological parameters on the accumulation of different compounds and provide important information for further studies of the relationship between wine production and climate adaptability. Future studies could further investigate the relationship between soil microbial communities and the synthesis of grape volatile compounds in different producing areas, which would provide an important scientific basis for optimizing grape planting strategies and addressing climate change, thereby ensuring the flavor stability and quality of wine.

## 4. Conclusions

In general, this study achieved the effective identification of different sub-producing areas at the eastern foothills of Helan Mountain through the combination of GC×GC-TOFMS technology and a chemometrics analysis. Moreover, it revealed the influence of climatic factors on flavor characteristics, thereby providing important theoretical support for addressing the homogenization issue of wine in small producing areas and establishing a traceability technology system for producing areas. It also offered a new perspective on the sustainable development of the wine industry. A total of 184 volatile compounds were identified, of which 124 were common to all producing areas. Although some specific compounds were only present in specific producing areas, these compounds did not play a crucial role in the identification of producing areas. This reflects that the main aroma characteristics do not simply depend on the presence of a few volatile compounds. The main volatile components were fermentation-derived alcohols and esters, which endow wine with aromatic properties. Through identifying compounds with an OAV > 1 and conducting an aroma vector analysis, it was discovered that “flowery” and “fruity” are the most important aromatic series in Cabernet Sauvignon wines from the eastern foothills of Helan Mountain. Moreover, relatively lower maturity can enhance the retention of these desirable aromatic characteristics. Fifteen substances (VIP > 1) play a crucial role in the identification of producing areas, particularly phenylethyl alcohol and isoamyl alcohol. The accumulation of phenylethyl alcohol was negatively correlated with the duration of sunshine during the growth stage, while it was significantly positively correlated during the maturity stage. Due to the crucial role of phenylethyl alcohol in the identification of producing areas, the impact of sunshine conditions on the variation in wine flavor was further demonstrated. Wine producers can adjust the harvesting time based on the climate data during the growth stage to optimize the flavor characteristics. Future studies can further investigate the underlying biological mechanisms of the observed correlations, particularly the effects of environmental factors on the accumulation of volatile compounds.

## Figures and Tables

**Table 1:** Scaling curves of volatile compounds.

Aroma Category	CAS	Chemical Formula	Name	Configure Concentration Range	Standard Curve	Linearly Dependent Coefficient
C_6_ Compounds	111-27-3	C_6_H_14_O	Hexanol	100–1600 μg/L	y = 1.2458x − 0.0375	R^2^ = 0.9643
	66-25-1	C_6_H_12_O	Hexanal	10–160 μg/L	y = 8.0136x + 0.011	R^2^ = 0.9997
	928-95-0	C_6_H_12_O	(E)-2-Hexenol	10–160 μg/L	y = 2.4937x − 0.009	R^2^ = 0.9986
	928-94-9	C_6_H_12_O	(Z)-2-Hexenol	10–160 μg/L	y = 2.6556x − 0.0033	R^2^ = 0.9981
	6728-26-3	C_6_H_10_O	(E)-2-Hexenal	100–1600 μg/L	y = 3.3488x + 0.8776	R^2^ = 0.9972
	142-83-6	C_6_H_8_O	(E, E)-2,4-Hexadienal	10–160 μg/L	y = 5.3355x − 0.0463	R^2^ = 0.9946
Alcohols	111-87-5	C_8_H_18_O	Octanol	10–160 μg/L	y = 51.698x − 0.5564	R^2^ = 0.9957
	143-08-8	C_9_H_20_O	1-Nonanol	10–160 μg/L	y = 71.56x − 1.2978	R^2^ = 0.9913
	123-96-6	C_8_H_18_O	2-Octanol	10–160 μg/L	y = 35.682x − 0.5183	R^2^ = 0.9861
	3391-86-4	C_8_H_16_O	1-Octen-3-ol	10–160 μg/L	y = 24.777x − 0.1763	R^2^ = 0.9982
	111-70-6	C_7_H_16_O	1-Heptanol	10–160 μg/L	y = 22.935x − 0.1722	R^2^ = 0.9963
	123-51-3	C_5_H_12_O	3-Methyl-1-butanol	10–160 μg/L	y = 4.2804x − 0.1309	R^2^ = 0.9473
	626-89-1	C_6_H_14_O	4-Methyl-1-pentanol	10–160 μg/L	y = 2.1821x + 0.2708	R^2^ = 0.9911
	104-76-7	C_8_H_18_O	2-Ethylhexanol	10–160 μg/L	y = 52.137x − 0.4486	R^2^ = 0.9967
	60-12-8	C_8_H_10_O	Phenethyl alcohol	10–160 μg/L	y = 4.4301x − 0.2658	R^2^ = 0.9537
	589-98-0	C_8_H_18_O	3-Octanol	10–160 μg/L	y = 38.532x + 0.8656	R^2^ = 0.9944
	100-51-6	C_7_H_8_O	Benzyl alcohol	10–160 μg/L	y = 3.3507x − 0.1344	R^2^ = 0.9955
	137-32-6	C_5_H_12_O	2-Methyl-1-butanol	100–1600 μg/L	y = 7.0677x − 0.0259	R^2^ = 0.9991
	543-49-7	C_7_H_16_O	2-Heptanol	10–160 μg/L	y = 5.8322x + 0.446	R^2^ = 0.9918
Esters	123-86-4	C_6_H_12_O_2_	Butyl acetate	10–160 μg/L	y = 6.2698x − 0.0748	R^2^ = 0.9852
	142-92-7	C_8_H_16_O_2_	Hexyl acetate	10–160 μg/L	y = 77.214x − 2.5275	R^2^ = 0.9922
	119-36-8	C_8_H_8_O_3_	Methyl salicylate	10–160 μg/L	y = 33.87x − 1.4589	R^2^ = 0.9913
	93-58-3	C_8_H_8_O_2_	Methyl benzoate	10–160 μg/L	y = 43.754x − 1.3116	R^2^ = 0.9918
	141-32-2	C_7_H_12_O_2_	Butyl acrylate	10–160 μg/L	y = 42.2x − 0.8667	R^2^ = 0.9926
	141-78-6	C_4_H_8_O_2_	Ethyl acetate	100–1600 μg/L	y = 0.027x − 0.0002	R^2^ = 0.9964
	105-54-4	C_6_H_12_O_2_	Ethyl butyrate	10–160 μg/L	y = 29.542x − 0.6768	R^2^ = 0.9914
	123-92-2	C_7_H_14_O_2_	Isoamyl acetate	10–160 μg/L	y = 36.549x − 0.6111	R^2^ = 0.9927
	123-66-0	C_8_H_16_O_2_	Ethyl hexanoate	10–160 μg/L	y = 163.94x − 3.9053	R^2^ = 0.9961
	106-32-1	C_10_H_20_O_2_	Ethyl caprylate	10–160 μg/L	y = 229.85x − 6.9287	R^2^ = 0.9948
	123-25-1	C_8_H_14_O_4_	Diethyl succinate	10–160 μg/L	y = 13.16x − 0.5471	R^2^ = 0.9915
	111-11-5	C_9_H_18_O_2_	Caprylic acid methyl ester	10–160 μg/L	y = 179.14x − 6.1591	R^2^ = 0.9921
	638-11-9	C_7_H_14_O_2_	Isopropyl butyrate	10–160 μg/L	y = 13.046x − 0.3695	R^2^ = 0.9948
	624-41-9	C_7_H_14_O_2_	2-Methylbutyl acetate	100–1600 μg/L	y = 102.51x − 0.0826	R^2^ = 0.9971
	105-66-8	C_7_H_14_O_2_	Propyl butyrate	10–160 μg/L	y = 54.77x − 0.8899	R^2^ = 0.996
	37064-20-3	C_8_H_16_O_2_	Natural propyl 2-methylbutyrate	10–160 μg/L	y = 86.527x − 2.316	R^2^ = 0.9949
	112-06-1	C_9_H_18_O_2_	Heptyl acetate	10–160 μg/L	y = 142.01x − 5.8118	R^2^ = 0.9916
	623-42-7	C_5_H_10_O_2_	Methyl butyrate	10–160 μg/L	y = 10.822x + 0.6266	R^2^ = 0.9983
	868-57-5	C_6_H_12_O_2_	Methyl 2-methylbutyrate	10–160 μg/L	y = 13.384x − 0.1074	R^2^ = 0.9969
	7452-79-1	C_7_H_14_O_2_	Ethyl 2-methylbutyrate	10–160 μg/L	y = 67.904x − 0.5643	R^2^ = 0.9972
	15706-73-7	C_9_H_18_O_2_	Butyl 2-methylbutanoate	10–160 μg/L	y = 167.71x − 6.1318	R^2^ = 0.9943
Aldehydes	111-71-7	C_7_H_14_O	Heptanal	10–160 μg/L	y = 29.74x + 0.0397	R^2^ = 0.9982
	124-13-0	C_8_H_16_O	Octanal	10–160 μg/L	y = 125.84x − 2.0365	R^2^ = 0.9934
	124-19-6	C_9_H_18_O	Nonanal	10–160 μg/L	y = 28.958x − 0.0203	R^2^ = 0.9972
	100-52-7	C_7_H_6_O	Benzaldehyde	10–160 μg/L	y = 12.281x − 0.1913	R^2^ = 0.9974
	18829-55-5	C_7_H_12_O	(E)-2-Heptenal	10–160 μg/L	y = 23.382x + 0.1089	R^2^ = 0.9989
	2548-87-0	C_8_H_14_O	(E)-2-Octenal	10–160 μg/L	y = 66.898x − 1.1385	R^2^ = 0.9975
	4313-03-5	C_7_H_10_O	(E, E)-2,4-Heptadienal	10–160 μg/L	y = 16.991x − 0.1225	R^2^ = 0.9996
	5910-87-2	C_9_H_14_O	(E, E)-2,4-Nonadienal	10–160 μg/L	y = 32.147x − 0.9573	R^2^ = 0.9921
	112-31-2	C_10_H_20_O	Decanal	10–160 μg/L	y = 259.53x − 10.679	R^2^ = 0.9923
	18829-56-6	C_9_H_16_O	(E)-2-Nonenal	10–160 μg/L	y = 145.84x − 5.5835	R^2^ = 0.9927
	557-48-2	C_9_H_14_O	(E, Z)-2,6-Nonadienal	10–160 μg/L	y = 47.523x − 1.0794	R^2^ = 0.9998
Terpenes	99-83-2	C_10_H_16_	*α*-Phellandrene	10–160 μg/L	y = 152.44x − 3.4687	R^2^ = 0.9985
	123-35-3	C_10_H_16_	*β*-Myrcene	10–160 μg/L	y = 141.09x − 2.865	R^2^ = 0.9971
	106-25-2	C_10_H_18_O	Nerol	10–160 μg/L	y = 35.863x − 0.6729	R^2^ = 0.9937
	16409-43-1	C_10_H_18_O	(Z)-Rose oxide	10–160 μg/L	y = 209.19x − 4.503	R^2^ = 0.9982
	78-70-6	C_10_H_18_O	Linalool	10–160 μg/L	y = 79.016x − 0.1611	R^2^ = 0.9941
	562-74-3	C_10_H_18_O	4-Terpinenol	10–160 μg/L	y = 69.579x − 0.3551	R^2^ = 0.9995
	98-55-5	C_10_H_18_O	*α*-Terpineol	10–160 μg/L	y = 46.539x − 0.6597	R^2^ = 0.9965
	106-22-9	C_10_H_20_O	Citronellol	10–160 μg/L	y = 67.36x − 1.497	R^2^ = 0.9972
	106-24-1	C_10_H_18_O	Geraniol	10–160 μg/L	y = 29.682x − 0.3935	R^2^ = 0.9939
	87-44-5	C_15_H_24_	*β*-Caryophyllene	10–160 μg/L	y = 189.71x − 8.1006	R^2^ = 0.9924
	536-59-4	C_10_H_16_O	Dihydro cuminyl alcohol	10–160 μg/L	y = 11.437x − 0.2996	R^2^ = 0.9951
	99-49-0	C_10_H_14_O	Carvone	10–160 μg/L	y = 63.254x − 2.057	R^2^ = 0.9951
	5989-27-5	C_10_H_16_	D-Limonene	10–160 μg/L	y = 189.67x − 1.6892	R^2^ = 0.9928
	99-87-6	C_10_H_14_	*p*-Cymene	10–160 μg/L	y = 171.9x − 1.0382	R^2^ = 0.9967
	502-61-4	C_15_H_24_	Farnesene	10–160 μg/L	y = 69.691x − 3.4768	R^2^ = 0.9871
C_13_-Norisoprenoids	23696-85-7	C_13_H_18_O	*β*-Damascenone	10–160 μg/L	y = 148.1x − 3.6408	R^2^ = 0.9911
	689-67-8	C_13_H_22_O	Geranylacetone	10–160 μg/L	y = 276.85x − 8.7723	R^2^ = 0.9926
	79-77-6	C_13_H_20_O	β-Ionone	10–160 μg/L	y = 88.038x − 1.3341	R^2^ = 0.9989
	4312-99-6	C_8_H_14_O	1-Octen-3-one	10–160 μg/L	y = 48.416x − 0.3725	R^2^ = 0.9973
	98-86-2	C_8_H_8_O	Acetophenone	10–160 μg/L	y = 17.348x − 0.4572	R^2^ = 0.9925
	1604-28-0	C_8_H_12_O	6-Methyl-3,5-heptadiene-2-one	10–160 μg/L	y = 18.08x − 0.3982	R^2^ = 0.9974
	110-93-0	C_8_H_14_O	6-Methyl-5-hepten-2-one	10–160 μg/L	y = 26.734x + 0.4244	R^2^ = 0.9955

**Table 2:** Geographical locations and altitudes of vineyard.

Producing Area	Name	Altitude	Latitude	Longitude
HL	Sunshine winery	1199.8	38.7127	106.0671
	Chateau HaiYueRenHe	1174.8	38.7113	106.0754
	Domaine Charme	1195.4	39.7135	106.0702
	Jade Vineyard	1180.4	38.7238	106.0838
	Hejinzun Winery	1202.1	38.7269	106.0776
YC	Legacy Peak Estate	1151.6	38.4411	106.0002
	Chateau Baoshi	1171.5	38.5720	106.0254
	Chateau Lanny	1153.6	38.6526	106.0534
	Chateau Mihope	1183	38.6228	106.0182
	Yuanshi Vineyard	1192.4	38.5826	106.0145
YN	LiLan Winery	1198.9	38.2759	105.9642
	Chateau Greatwall Terroir	1211.6	38.3807	105.9543
	Fei Tswei winery	1147.1	38.3739	105.9874
	Xinhunbin Winery	1145	38.2405	106.0277
	Chateau Yuquan of Ning Xia State Farm	1142.6	38.2627	106.0426
QTX	Xige Estate	1232	38.0769	105.8448
	Zhongzexiban Winery	1230.1	38.0402	105.8397
	Chateau Modern	1208.5	38.0950	105.8657
	Sweet Dew Vineyard	1171.2	38.1270	105.9359
	Huangkou Winery	1184.6	38.0754	105.8978
	Longyu Estate	1205.28	38.0470	105.5407
HSP	Xingyu Winery	1480.1	37.3405	106.1685
	Roland Margo	1482.5	37.3176	106.1640
	Baoyuan Dadi Winery	1512.7	37.3052	106.1780
	Chateau J.L. Jiangyuan	1456.2	37.3395	106.1567
	Mingyu Winery	1454	37.3494	106.1587

**Table 3:** Basic physical and chemical properties of wine from each producing area.

Sample Number	Residual Sugar/(g/L)	Titratable Acid/(g/L)	pH	Alcohol/(%vol)
HL1	3.03 ± 0.06 c	5.31 ± 0.04 bc	3.54 ± 0.01 c	12.87 ± 0.03 c
HL2	4.13 ± 0.06 a	5.74 ± 0.01 a	3.55 ± 0.01 bc	13.72 ± 0.04 b
HL3	3.10 ± 0.10 c	5.22 ± 0.04 cd	3.60 ± 0.01 a	12.85 ± 0.00 c
HL4	2.87 ± 0.23 c	5.08 ± 0.17 d	3.60 ± 0.04 a	12.81 ± 0.01 c
HL5	3.73 ± 0.15 b	5.45 ± 0.02 b	3.59 ± 0.01 ab	14.10 ± 0.03 a
Average HL	3.37 ± 0.51 A	5.36 ± 0.24 AB	3.58 ± 0.03 AB	13.27 ± 0.56 A
YC1	3.67 ± 0.15 a	5.17 ± 0.03 d	3.48 ± 0.00 d	12.26 ± 0.02 b
YC2	3.13 ± 0.15 b	5.43 ± 0.04 c	3.55 ± 0.00 b	12.02 ± 0.02 c
YC3	2.83 ± 0.06 c	5.83 ± 0.02 a	3.46 ± 0.01 e	10.53 ± 0.03 d
YC4	3.20 ± 0.10 b	4.38 ± 0.01 e	3.63 ± 0.00 a	12.29 ± 0.06 b
YC5	3.17 ± 0.15 b	5.62 ± 0.05 b	3.50 ± 0.01 c	14.37 ± 0.03 a
Average YC	3.20 ± 0.30 A	5.29 ± 0.52 AB	3.52 ± 0.06 B	12.29 ± 1.27 BC
YN1	3.27 ± 0.15 b	5.32 ± 0.03 bc	3.60 ± 0.01 a	13.35 ± 0.02 b
YN2	3.87 ± 0.15 a	5.67 ± 0.03 a	3.50 ± 0.01 d	12.85 ± 0.04 c
YN3	3.10 ± 0.10 bc	5.36 ± 0.03 b	3.59 ± 0.01 a	13.95 ± 0.03 a
YN4	2.87 ± 0.06 c	4.94 ± 0.01 d	3.54 ± 0.01 b	12.48 ± 0.05 d
YN5	3.17 ± 0.06 b	5.27 ± 0.03 c	3.52 ± 0.01 c	12.29 ± 0.02 e
Average YN	3.25 ± 0.36 A	5.31 ± 0.24 AB	3.55 ± 0.04 AB	12.99 ± 0.63 AB
QTX1	3.07 ± 0.12 bc	5.41 ± 0.01 b	3.57 ± 0.01 d	12.51 ± 0.05 d
QTX2	3.17 ± 0.06 bc	4.97 ± 0.03 d	3.61 ± 0.00 c	13.50 ± 0.03 b
QTX3	3.40 ± 0.17 b	5.51 ± 0.02 a	3.52 ± 0.00 e	12.82 ± 0.04 c
QTX4	4.17 ± 0.12 a	4.44 ± 0.03 f	3.49 ± 0.01 f	11.86 ± 0.08 f
QTX5	2.90 ± 0.10 c	4.66 ± 0.03 e	3.63 ± 0.00 b	12.24 ± 0.01 e
QTX6	3.20 ± 0.17 bc	5.20 ± 0.01 c	3.74 ± 0.01 a	15.89 ± 0.11 a
Average QTX	3.32 ± 0.43 A	5.03 ± 0.40 B	3.59 ± 0.08 A	13.14 ± 1.37 AB
HSP1	3.07 ± 0.06 bc	5.21 ± 0.04 d	3.41 ± 0.00 d	12.23 ± 0.03 b
HSP2	3.10 ± 0.00 bc	5.52 ± 0.02 b	3.47 ± 0.01 a	10.73 ± 0.01 d
HSP3	2.97 ± 0.15 c	5.94 ± 0.02 a	3.44 ± 0.00 c	12.41 ± 0.02 a
HSP4	3.20 ± 0.10 b	5.41 ± 0.03 c	3.45 ± 0.01 b	11.50 ± 0.02 c
HSP5	3.43 ± 0.06 a	5.91 ± 0.03 a	3.34 ± 0.01 e	12.42 ± 0.03 a
Average HSP	3.15 ± 0.18 A	5.60 ± 0.30 A	3.42 ± 0.05 C	11.86 ± 0.68 C

Note: Different lowercase letters represent significant differences between wine samples in the sub-producing areas (*p* < 0.05), and different uppercase letters represent significant differences between the producing areas (*p* < 0.05).

**Table 4:** Specific locations of meteorological stations in each production area.

Producing Area	Location of Weather Stations	Longitude	Latitude
HL	Hongguang Town, Helan County, Yinchuan City, Ningxia Hui Autonomous Region	106.05	38.75
	Hongguang Town, Helan County, Yinchuan City, Ningxia Hui Autonomous Region	106.15	38.75
	Hongguang Town, Helan County, Yinchuan City, Ningxia Hui Autonomous Region	106.05	38.85
YC	Beibao Town, Xixia District, Yinchuan City, Ningxia Hui Autonomous Region	106.05	38.65
	Beibao Town, Xixia District, Yinchuan City, Ningxia Hui Autonomous Region	106.05	38.55
	Beibao Town, Xixia District, Yinchuan City, Ningxia Hui Autonomous Region	105.95	38.65
YN	Huangyangtan Farm, Yongning County, Yinchuan City, Ningxia Hui Autonomous Region	106.05	38.45
	Huangyangtan Farm, Yongning County, Yinchuan City, Ningxia Hui Autonomous Region	106.05	38.35
	Huangyangtan Farm, Yongning County, Yinchuan City, Ningxia Hui Autonomous Region	105.95	38.35
QTX	Qujing Town, Qingtongxia City, Wuzhong City, Ningxia Hui Autonomous Region	105.95	38.15
	Daba Town, Qingtongxia City, Wuzhong City, Ningxia Hui Autonomous Region	105.95	38.05
	Daba Town, Qingtongxia City, Wuzhong City, Ningxia Hui Autonomous Region	105.85	38.05
HSP	Xinzhuangji Township, Hongsibu District, Wuzhong City, Ningxia Hui Autonomous Region	106.15	37.25
	Xinzhuangji Township, Hongsibu District, Wuzhong City, Ningxia Hui Autonomous Region	106.15	37.35
	Xinzhuangji Township, Hongsibu District, Wuzhong City, Ningxia Hui Autonomous Region	106.25	37.35

**Table 5:** Weather data of wine grape-producing areas in different phenological stages in 2023.

Meteorological Index	HL	YC	YN	QTX	HSP
Mean temperature of Apr (°C)	9.90	9.57	10.67	10.67	8.33
Mean temperature of May–Jun period (°C)	20.23	19.88	20.88	20.78	17.68
Mean temperature of Jul–Aug period (°C)	25.38	25.05	26.04	25.90	22.65
Mean temperature of Sept (°C)	20.15	19.78	20.69	20.53	17.45
Mean relative humidity of Apr (%)	36.13	36.21	36.58	37.14	40.42
Mean relative humidity of May–Jun period (%)	33.62	33.71	34.42	35.24	40.20
Mean relative humidity of Jul–Aug period (%)	38.20	38.06	38.61	39.33	44.78
Mean relative humidity of Sept (%)	42.94	43.26	44.83	46.38	53.94
Mean precipitation of Apr (mm)	11.02	12.07	14.43	14.70	19.74
Mean precipitation of May–Jun period (mm)	20.46	18.41	18.75	19.40	23.81
Mean precipitation of Jul–Aug period (mm)	23.82	26.37	26.25	31.81	43.40
Mean precipitation of Sept (mm)	29.03	26.36	22.00	25.18	29.33
Sunshine duration of Apr (h)	171.98	171.14	175.58	177.56	187.51
Sunshine duration of May–Jun period (h)	211.67	210.28	214.17	214.53	215.71
Sunshine duration of Jul–Aug period (h)	209.89	208.46	212.49	213.97	220.32
Sunshine duration of Sept (h)	157.65	154.11	154.43	152.27	157.43

Note: Correspondence between grape growth phenological period and months: germination period (April), flowering period (May to June), coloring period (July to August), and maturation period (September).

**Table 6:** Odor activity value (OAV > 1) and aroma type of volatile compounds.

NO.	CAS	Name	Threshold (μg/L)	Aroma Description	Type	HL	YC	YN	QTX	HSP
1	[104-67-6]	Undecan-4-olide	2.1 [46] a	Fruity, creamy	fruity	4.69	4.69	4.84	4.71	4.71
2	[105-54-4]	Ethyl butyrate	20 [47] b	Apple, pineapple	fruity	1.70	1.75	2.39	1.72	2.17
3	[105-68-0]	Isoamyl propionate	8.6 [48] a	Tropical fruit	fruity	1.46	1.44	1.60	1.54	1.65
4	[106-30-9]	Ethyl heptanoate	18 [49] c	Fruity, cognac, rum	fruity	0.99	1.00	1.00	1.03	1.22
5	[106-32-1]	Ethyl caprylate	5 [47] b	Fruity	waxy	29.24	34.24	29.38	23.21	39.27
6	[106-33-2]	Ethyl laurate	20 [50] b	Sweet, waxy, flower	waxy	1.94	2.15	1.13	1.41	1.46
7	[106-70-7]	Methyl hexanoate	10 [51] a	Fruity, bacon	fruity	1.13	1.21	1.33	1.18	1.35
8	[108-64-5]	Ethyl isovalerate	3 [49] c	Fruity, sweet, apple, pineapple	fruity	4.93	4.33	5.73	5.39	6.68
9	[110-19-0]	Isobutyl acetate	40 [52] d	Fruity odor, mild characteristic ester flavor	fruity	0.58	1.18	1.33	0.70	0.78
10	[111-27-3]	1-Hexanol	1100 [53] c	Gentle sweetness	herbal	1.02	1.15	1.05	0.97	1.73
11	[111-82-0]	Methyl laurate	1.5 [54] e	Wax, soapy, coconut, mushroom	waxy	7.70	7.60	6.77	6.92	7.18
12	[112-12-9]	2-Undecanone	5.5 [55] a	Fruity flavor	fruity	2.19	2.12	2.07	2.14	2.15
13	[112-31-2]	Decanal	1.25 [56] f	Pleasant smell	solvent	11.10	11.13	11.36	0.00	0.00
14	[112-54-9]	Dodecanal	0.29 [57] a	Flower fragrance	solvent	47.20	47.21	48.04	47.34	47.98
15	[116-53-0]	2-methyl-Butanoic acid	100 [58] a	Spicy, sour, goat milk cheese	solvent	1.88	1.72	1.26	1.78	1.83
16	[122-78-1]	Benzeneacetaldehyde	1 [59] f	Hyacinths, green	green	11.56	10.83	12.20	12.61	9.73
17	[123-66-0]	Ethyl Hexanoate	5 [60] c	Sweet, fruity	fruity	16.44	19.37	19.41	15.27	27.25
18	[123-92-2]	Isoamyl acetate	30 [50] b	Banana flavor	fruity	7.38	9.84	11.10	7.71	12.18
19	[124-06-1]	Ethyl myristate	2 [52] b	Violet	waxy	7.35	7.70	6.13	6.27	6.50
20	[124-13-0]	Octanal	2.5 [56] f	Fat, citrus	solvent	2.17	0.00	2.20	2.19	2.05
21	[124-19-6]	Nonanal	1 [56] f	Cured, peas	solvent	4.07	4.43	4.43	4.52	5.19
22	[18829-56-6]	(E)-2-Nonenal	0.6 [56] f	Fat, cucumber, aldehydes, citrus	fatty	20.90	0.00	0.00	0.00	20.97
23	[23726-93-4]	*β*-Damascenone	0.05 [52] g	Apple, rose, honey, tobacco, sweet	fruity	245.43	279.35	278.00	239.98	305.44
24	[35854-86-5]	(6Z)-Nonen-1-ol	1 [61] a	Cucumber	green	8.63	7.28	7.75	8.12	9.20
25	[3777-69-3]	2-Pentyl-Furan	5.8 [55] a	Fruity flavor	fruity	1.81	1.78	1.75	1.77	1.81
26	[432-25-7]	Beta-cyclocitral	3 [62] a	Herbs, rose oxide, tobacco, fruity	herbal	4.49	4.48	4.49	4.48	4.52
27	[5405-41-4]	Ethyl 3-hydroxybutyrate	20 [47] b	Fruity, grapes	fruity	0.77	0.83	0.00	0.72	1.18
28	[56805-23-3]	(E, Z)-3,6-Nonadien-1-ol	3 [61] a	Fruity	green	2.18	0.00	2.16	2.16	0.00
29	[6066-49-5]	3-N-Butylphthalide	10 [63] a	Herbs, phenol, celery	herbal	1.00	1.00	1.01	1.00	1.02
30	[64187-83-3]	Ethyl 3Z-Hexenoate	10 [64] e	Apple	green	1.55	1.60	1.50	1.64	1.79
31	[706-14-9]	*γ*-Decalactone	0.7 [65] b	Coconut butter, sweet	fruity	0.00	0.00	14.11	14.16	0.00
32	[7452-79-1]	Ethyl 2-methylbutyrate	2 [66] c	pungent, green apple, fruity	fruity	3.01	2.71	3.17	2.83	3.38
33	[79-77-6]	trans-*β*-Ionone	0.007 [51] a	Flowers, berries	floral	760.12	770.81	771.37	752.16	813.99
34	[821-55-6]	2-Nonanone	10.9 [67] a	Soil, herb	fruity	1.02	1.01	1.15	1.12	1.21
35	[88-29-9]	Versalide	2.4 [68] a	Musk	musk	4.34	4.32	4.33	4.33	4.35
36	[97-62-1]	Ethyl isobutyrate	15 [69] b	Wine	fruity	1.23	0.81	1.03	1.61	1.73

Note: Substrate of odor threshold: water solution at pH 2.0 (a); 14%vol ethanol/water (*v/v*) solution (b); 12% ethanol/water (*v/v*) solution at pH 3.5 (c); 10% ethanol/water (*v/v*) solution at pH 3.2 (d); air (e); 10 % water/ethanol, tartaric acid 5 g/L, pH 3.2 (f); wine (g).

## Data Availability

The data presented in this study are available upon request from the corresponding authors. The data are not publicly available due to privacy restrictions.

## Supplementary Materials

The following supporting information can be downloaded at: https://www.mdpi.com/article/10.3390/foods13223644/s1, Table S1: The content of volatile compounds in wines from different producing areas.
