# Supplementary material for: Aroma Identification and Traceability of the Core Sub-Producing Area in the Helan Mountain Eastern Foothills Using Two-Dimensional Gas Chromatography and Time-of-Flight Mass Spectrometry and Chemometrics
Source: Foods. 2024 Nov 15;13(22):3644. doi: 10.3390/foods13223644 (PMC11594276; doi:10.3390/foods13223644)
Supplement: Supplementary file 1 [file foods-13-03644-s001.zip › foods-3282488-supplementary.pdf]

Supplementary Materials:

Table S1. The content of volatile compounds in wines from different producing areas

ID	CAS	Name	^{1}tR (s)	^{2}tR (s)	RI	NIST-RI	HL	YC	YN	QTX	HSP
	Alcohols										
A1	[100-51-6]	Benzyl alcohol	37.4522	0.475	1873	1870	159.81±1.51b	123.01±1.35c	107.99±5.81d	201.26±0.51a	123.81±2.75c
A2	[104-76-7]	2-Ethyl-1-hexanol	23.1815	1.065	1488	1491	4.10±0.04b	4.29±0.14b	5.44±0.44a	5.38±0.37a	5.12±0.28a
A3	[111-70-6]	1-Heptanol	21.8498	0.885	1455	1453	31.97±0.23d	35.20±0.30c	39.10±1.82b	30.17±0.93d	42.58±0.65a
A4	[111-87-5]	1-Octanol	25.8494	0.971	1556	1557	12.31±0.32c	13.24±0.40bc	18.67±0.94a	14.37±0.53b	17.53±0.10a
A5	[112-30-1]	1-Decanol	33.5164	1.090	1761	1760	12.25±0.22a	9.17±0.34c	-	10.29±0.39b	10.42±0.23b
A6	[112-53-8]	1-Dodecanol	40.5831	1.199	1966	1969	12.60±0.15b	13.03±0.08b	12.71±0.49b	11.40±0.48c	16.40±0.77a
A7	[123-51-3]	3-Methyl-1-butanol	12.8510	1.471	1224	1209	3485.27±11.17b	3554.15±11.39a	2715.32±26.96d	2680.34±25.00d	3369.02±29.63c
A8	[126-30-7]	Neopentyl glycol	9.1192	0.495	1109	1109	-	121.35±33.22a	-	25.50±1.51b	-
A9	[126-86-3]	2,4,7,9-Tetramethyl-5-decyn-4,7-diol	44.7162	0.819	2096	2094	-	7.64±0.12	-	-	-
A10	[13019-22-2]	9-Decen-1-ol	35.3857	0.816	1814	1813	8.17±0.20ab	7.89±0.09b	8.28±0.02a	8.08±0.07ab	7.93±0.09b
A11	[143-08-8]	1-Nonanol	29.7166	1.010	1657	1660	14.53±0.24b	15.08±0.06b	14.51±0.65b	14.31±0.18b	16.61±0.06a
A12	[1565-80-6]	(S)-(-)-2-Methyl-1-butanol	12.4497	0.945	1213	1211	300.75±3.28a	219.48±8.23b	187.95±3.16d	206.92±1.27c	-
A13	[35854-86-5]	(6Z)-Nonen-1-ol	31.8497	0.959	1715	1711	8.63±0.19b	7.28±0.23d	7.75±0.13cd	8.12±0.11c	9.20±0.37a
A14	[36653-82-4]	1-Hexadecanol	47.1180	1.245	2382	2382	11.36±0.06b	10.08±0.36cd	10.60±0.40c	9.80±0.20d	13.75±0.19a
A15	[40716-66-3]	Nerolidol	42.8496	1.450	2037	2042	8.80±0.06a	8.56±0.02b	8.31±0.01c	8.05±0.08d	9.03±0.22a
A16	[4602-84-0]	Farnesyl	52.4500	1.205	2350	2350	8.03±0.16a	7.51±0.04c	7.51±0.05c	7.43±0.07c	7.77±0.05b
A17	[51411-24-6]	(+/-) Dihydrafarnesol	49.9145	1.378	2270	2270	7.98±0.16ab	7.78±0.07ab	7.85±0.13ab	7.70±0.08b	8.08±0.16a
A18	[56805-23-3]	(E, Z)-3,6-Nonadien-1-ol	32.4523	0.798	1732	1747	6.53±0.30a	-	6.47±0.20a	6.48±0.14a	-
A19	[589-35-5]	3-Methyl-1-pentanol	16.8498	0.798	1329	1326	181.94±3.61a	147.14±1.99b	147.01±1.06b	117.70±0.24c	143.97±0.86b
A20	[60-12-8]	Phenylethyl alcohol	38.7166	0.735	1910	1907	5531.87±9.76a	4731.61±37.14c	4131.32±20.17d	4037.59±45.99e	4843.05±10.06b
A21	[71-41-0]	1-Pentanol	13.8511	0.602	1250	1250	39.43±0.24b	77.22±2.65a	-	-	14.46±0.92c
A22	[77-53-2]	Cedrol	45.3829	1.804	2116	2116	7.67±0.02ab	7.43±0.02c	7.51±0.07bc	7.41±0.06c	7.73±0.13a
A23	[78-83-1]	2-Methyl-1-propanol	9.0523	0.672	1107	1092	62.65±0.47a	-	31.06±1.39c	28.18±3.91c	40.18±1.39b
A24	[821-09-0]	4-Penten-1-ol	14.1807	0.840	1259	1299	-	-	-	41.72±0.14	-
A25	[589-98-0]	3-Octanol	19.3190	0.924	1392	1393	5.06±0.13a	-	5.03±0.04a	5.02±0.06a	5.11±0.05a

A26	[5944-20-7]	Iso-Geraniol	35.2496	0.986	1810	1820	7.18±0.12bc	6.95±0.07c	7.29±0.08b	6.91±0.06c	7.55±0.19a
	Subtotal						9721.18±16.89a	8768.67±43.60b	7368.87±5.89d	7373.87±35.43d	8678.07±30.04c
	Esters										
B1	[101-97-3]	Ethyl phenylacetate	34.1833	1.361	1780	1783	11.48±0.23ab	11.22±0.17b	11.45±0.10ab	11.26±0.05b	11.82±0.16a
B2	[103-26-4]	Methyl cinnamate	43.9176	1.092	2070	2054	10.01±0.20	-	-	-	-
B3	[103-45-7]	Phenethyl acetate	35.3110	1.616	1812	1813	19.86±0.17b	21.99±0.47a	21.60±0.39a	17.47±0.45c	19.90±0.22b
B4	[103-48-0]	Phenethyl isobutyrate	40.3857	1.440	1960	1896	10.25±0.05a	10.08±0.11b	10.00±0.10b	10.00±0.03b	-
B5	[10348-47-7]	Ethyl 2-hydroxy-4-methylvalerate	25.1831	1.008	1539	1547	12.38±0.14cd	12.21±0.10d	13.06±0.16b	12.55±0.15c	15.13±0.06a
B6	[103-52-6]	Β-Phenylethyl butyrate	40.3829	1.586	1960	1958	10.17±0.08a	10.15±0.10a	10.16±0.07a	9.99±0.01b	10.04±0.02ab
B7	[104-61-0]	γ-Nonanolactone	42.4498	1.179	2024	2024	13.78±0.04b	13.61±0.02c	13.78±0.06b	13.72±0.04bc	13.94±0.09a
B8	[104-62-1]	Phenethyl formate	34.1846	1.023	1780	1771	13.62±0.04ab	13.57±0.02b	13.58±0.05b	13.68±0.06ab	13.78±0.12a
B9	[104-67-6]	Undecan-4-olide	46.0498	1.217	2259	2259	9.84±0.02b	9.84±0.01b	10.16±0.03a	9.89±0.04b	9.89±0.01b
B10	[105-54-4]	Ethyl butyrate	7.2497	1.180	1033	1036	34.10±0.33c	35.09±0.58c	47.84±1.02a	34.32±1.50c	43.37±0.51b
B11	[105-68-0]	Isoamyl propionate	10.9832	1.861	1169	1185	12.58±0.22c	12.42±0.01c	13.74±0.15ab	13.22±0.29b	14.17±0.37a
B12	[105-79-3]	Isobutyl hexanoate	17.2500	2.488	1340	1350	10.12±0.03c	10.23±0.03bc	10.28±0.03b	10.21±0.06bc	10.46±0.07a
B13	[106-27-4]	Isoamyl butyrate	14.0495	2.190	1256	1259	13.79±0.05bc	13.82±0.01bc	13.75±0.02c	14.03±0.07a	13.88±0.05b
B14	[106-30-9]	Ethyl heptanoate	16.5165	2.169	1321	1331	17.87±0.17b	18.06±0.17b	17.95±0.18b	18.59±0.22b	22.02±0.63a
B15	[106-32-1]	Ethyl caprylate	20.7164	2.374	1427	1435	146.21±0.80c	171.22±3.31b	146.89±2.53c	116.03±3.69d	196.33±3.77a
B16	[106-33-2]	Ethyl laurate	36.2498	2.558	1839	1843	38.88±0.58b	43.06±0.71a	22.55±0.11d	28.29±0.74c	29.13±0.35c
B17	[106-70-7]	Methyl hexanoate	10.9164	1.604	1167	1184	11.32±0.17c	12.07±0.11b	13.27±0.53a	11.84±0.10bc	13.54±0.27a
B18	[108-64-5]	Ethyl isovalerate	7.9164	1.368	1062	1068	14.80±0.40d	12.98±0.00e	17.20±0.46b	16.18±0.34c	20.03±0.51a
B19	[109-25-1]	Heptanoic acid 3-methylbutyl ester	25.3845	2.679	1545	1550	-	9.87±0.04a	9.83±0.01a	9.82±0.01a	9.85±0.02a
B20	[110-19-0]	Isobutyl acetate	6.7165	1.067	1011	1012	23.32±0.12e	47.02±1.57b	53.16±1.07a	28.18±0.26d	31.33±0.45c
B21	[110-27-0]	Isopropyl myristate	42.783	2.898	2035	2027	11.96±0.22b	11.30±0.06b	10.78±0.16b	11.43±0.55b	14.54±0.85a
B22	[110-38-3]	Decanoic acid ethyl ester	28.7166	2.498	1631	1639	126.63±0.49ab	138.10±1.75a	87.82±11.17c	79.46±0.64c	116.97±1.27b
B23	[110-42-9]	Decanoic acid methyl ester	27.0500	2.200	1587	1593	12.03±0.17ab	12.22±0.19a	12.40±0.20a	11.64±0.19b	12.00±0.06ab
B24	[111-11-5]	Octanoic acid methyl ester	18.7833	2.009	1379	1385	13.29±0.03d	13.68±0.25c	14.50±0.21a	13.86±0.13bc	14.21±0.13ab
B25	[111-61-5]	Octadecanoic acid ethyl ester	55.3166	2.786	2451	2451	10.37±0.03a	10.23±0.09a	10.17±0.10a	10.22±0.07a	10.35±0.10a
B26	[111-62-6]	Ethyl Oleate	55.8498	2.650	2471	2471	10.97±0.06a	10.58±0.11b	10.41±0.10b	10.62±0.05b	10.57±0.08b
B27	[1117-55-1]	Octanoic acid hexyl ester	35.1189	2.620	1807	1796	9.85±0.01ab	9.87±0.01ab	9.83±0.02b	-	9.88±0.03a

B28	[111-82-0]	Methyl laurate	34.7831	2.324	1797	1804	11.54±0.12a	11.40±0.27a	10.16±0.06c	10.37±0.03bc	10.78±0.26b
B29	[112-06-1]	Acetic acid heptyl ester	18.1831	1.970	1363	1377	13.71±0.03b	14.16±0.08a	14.30±0.05a	14.02±0.10a	14.10±0.24a
B30	[112-14-1]	Acetic acid octyl ester	22.0512	2.063	1461	1475	-	10.66±0.17a	10.24±0.06b	10.55±0.24ab	10.50±0.17ab
B31	[112-39-0]	Methyl palmitate	48.3831	2.500	2209	2209	11.38±0.25b	11.52±0.31b	10.92±0.05b	11.12±0.40b	12.61±0.25a
B32	[112-61-8]	Methyl stearate	54.3141	2.716	2418	2418	-	9.88±0.06b	9.93±0.05b	10.07±0.03a	9.92±0.00b
B33	[118-60-5]	2-Ethylhexyl salicylate	50.6477	1.962	2305	2305	9.95±0.06c	10.13±0.09b	-	-	10.38±0.10a
B34	[118-61-6]	Ethyl 2-hydroxybenzoate	34.9809	1.492	1802	1820	15.38±0.17b	14.50±0.16c	-	17.62±0.22a	15.44±0.51b
B35	[1189-09-9]	trans-Geranic acid methyl ester	30.9149	1.950	1690	1677	9.92±0.01b	9.90±0.01b	9.85±0.02c	9.99±0.02a	9.90±0.02b
B36	[119-36-8]	Methyl salicylate	33.7831	1.216	1769	1765	17.69±0.18b	18.01±0.14b	17.88±0.41b	28.07±0.38a	17.61±0.33b
B37	[122-70-3]	2-Phenethyl propionate	37.6478	1.576	1879	1879	3.60±6.23ab	10.21±0.28a	-	10.13±0.07a	10.03±0.17a
B38	[123-25-1]	Diethyl succinate	30.2499	1.269	1672	1681	108.96±0.87b	98.64±1.36c	107.11±1.12b	83.09±1.09d	130.52±1.31a
B39	[123-29-5]	Ethyl nonanoate	24.7167	2.401	1528	1532	13.69±0.16a	13.26±0.18b	11.94±0.13d	12.28±0.20cd	12.55±0.05c
B40	[123-66-0]	Ethyl Hexanoate	12.5830	1.956	1217	1233	82.22±0.97c	96.83±2.00b	97.03±2.26b	76.34±3.07d	136.26±1.67a
B41	[123-92-2]	Isoamyl acetate	9.1151	1.501	1110	1123	221.5±2.99e	295.31±3.94c	333.07±3.03b	231.39±3.65d	365.31±1.34a
B42	[124-06-1]	Ethyl myristate	43.1833	2.621	2048	2050	14.69±0.24a	15.41±1.10a	12.26±0.13b	12.55±0.25b	13.00±0.58b
B43	[124-10-7]	Methyl tetradecanoate	41.9148	2.487	2007	2006	10.05±0.09a	10.02±0.06a	9.99±0.02a	9.95±0.06a	10.09±0.07a
B44	[131-11-3]	Dimethyl phthalate	50.6477	1.133	2303	2303	-	9.93±0.11a	9.97±0.05a	10.02±0.11a	10.08±0.15a
B45	[140-11-4]	Benzyl acetate	32.1831	1.206	1724	1720	14.09±0.10a	13.73±0.07b	13.66±0.14b	13.58±0.07bc	13.44±0.02c
B46	[141-32-2]	2-Propenoic acid butyl ester	10.8533	1.259	1165	1184	-	7.22±0.03	-	-	-
B47	[142-91-6]	Isopropyl palmitate	49.0498	2.970	2235	2235	10.23±0.28ab	9.98±0.07b	10.14±0.18ab	9.93±0.07b	10.43±0.12a
B48	[142-92-7]	Acetic acid hexyl ester	14.1166	1.790	1258	1273	15.31±0.32d	22.80±0.57b	27.81±0.20a	18.22±0.68c	27.04±0.20a
B49	[143-13-5]	Acetic acid nonyl ester	26.1842	2.122	1565	1581	-	9.90±0.02a	9.83±0.01a	-	9.90±0.10a
B50	[15399-05-0]	Ethyl 2-hydroxy-3-phenylpropanoate	50.1167	0.886	2277	2277	10.65±0.15b	10.90±0.18b	10.94±0.15b	10.64±0.10b	11.51±0.23a
B51	[1552-67-6]	ethyl hex-2-enoate	17.1166	1.811	1336	1340	43.79±0.68c	46.21±1.25c	55.70±1.66b	48.85±1.44c	85.12±5.58a
B52	[16409-44-2]	Geranyl acetate	33.1165	1.877	1751	1751	9.84±0.03b	10.24±0.17a	9.86±0.01b	9.90±0.02b	9.93±0.04b
B53	[2021-28-5]	Ethyl 3-phenylpropionate	37.6497	1.441	1879	1893	10.83±0.13a	10.26±0.02bc	10.39±0.12b	10.17±0.02c	10.38±0.06b
B54	[2035-99-6]	Isoamyl caprylate	29.5164	2.794	1653	1658	16.12±0.24a	15.97±0.70a	13.04±0.22c	14.22±0.20b	15.66±0.15a
B55	[2050-01-3]	Isopentyl isobutyrate	11.0498	2.216	1172	1192	13.59±0.11b	13.53±0.06b	13.77±0.16b	13.66±0.04b	14.30±0.26a
B56	[2198-61-0]	Isopentyl hexanoate	21.5833	2.634	1449	1451	14.55±0.12a	14.80±0.27a	13.41±0.07b	13.46±0.22b	14.84±0.69a
B57	[2305-25-1]	Ethyl 3-hydroxybhexanoate	30.4524	0.819	1677	1674	-	-	11.56±0.19a	11.07±0.04b	-

B58	[2306-88-9]	Octanoic Acid octyl Ester	41.9817	2.908	2010	2009	9.86±0.01a	9.83±0.02a	9.96±0.12a	9.85±0.00a	9.89±0.06a
B59	[2306-91-4]	Pentadecanoic acid, 3-methylbutyl ester	36.9165	2.843	1859	1863	13.38±0.17b	14.57±0.27a	12.53±0.18c	12.23±0.32c	12.25±0.05c
B60	[2396-83-0]	3-Hexenoic acid ethyl ester	15.5831	1.685	1297	1290	16.88±0.61cd	16.21±0.33d	18.51±0.63b	17.69±0.73bc	20.96±0.40a
B61	[24851-98-7]	Methyl dihydrojasmonate	50.315	1.495	2264	2264	-	-	10.01±0.09a	9.94±0.06a	10.02±0.07a
B62	[2497-18-9]	trans-2-Hexenyl acetate	16.6524	1.438	1324	1333	-	14.69±0.58	-	-	-
B63	[28024-16-0]	Ethyl isopentyl succinate	38.2498	1.528	1897	1901	11.21±0.09ab	11.02±0.18bc	11.06±0.14abc	10.74±0.07c	11.45±0.25a
B64	[28267-29-0]	Ethyl tridecanoate	39.7831	2.603	1943	1947	10.20±0.11a	10.34±0.17a	10.05±0.04a	10.06±0.03a	10.23±0.15a
B65	[30673-38-2]	*n*-Capric acid isobutyl ester	33.0499	2.822	1749	1750	10.24±0.03b	10.52±0.02a	10.16±0.02c	10.10±0.01c	10.15±0.04c
B66	[30673-60-0]	Decanoic acid propyl ester	31.9165	2.647	1718	1724	10.05±0.07ab	10.09±0.03a	9.93±0.03c	9.92±0.03c	9.98±0.05bc
B67	[35194-38-8]	7-Octenoic acid ethyl ester	22.9113	2.237	1482	1478	12.53±0.23b	12.38±0.11bc	13.13±0.18a	13.10±0.23a	12.04±0.19c
B68	[3681-71-8]	cis-3-Hexenyl acetate	15.5833	1.614	1297	1316	-	-	-	15.01±0.14	-
B69	[41114-00-5]	Pentadecanoic acid ethyl ester	46.3778	2.989	2148	2148	-	-	10.28±0.01a	10.17±0.12a	-
B70	[4192-77-2]	Ethyl (E)-cinnamate	45.6500	1.241	2130	2130	10.04±0.03ab	10.08±0.05a	10.14±0.05a	9.97±0.01b	10.07±0.05ab
B71	[539-82-2]	Ethyl valerate	9.4484	1.662	1120	1134	7.27±0.30a	7.28±0.08a	7.86±0.20b	8.14±0.10b	9.79±0.29a
B72	[5405-41-4]	Ethyl 3-hydroxybutyrate	24.2483	0.891	1515	1515	15.40±1.20ab	16.57±0.13b	-	14.33±0.25c	23.61±0.31a
B73	[5444-75-7]	Benzoic acid 2-ethylhexyl ester	47.0495	1.950	2598	2598	10.27±0.05ab	9.98±0.02b	10.24±0.10ab	10.21±0.16b	10.56±0.22a
B74	[5461-06-3]	*n*-Caprylic acid isobutyl ester	25.3165	2.734	1543	1548	10.87±0.08b	11.43±0.12a	10.76±0.18bc	10.55±0.07c	10.86±0.13b
B75	[589-75-3]	Butyl caprylate	27.8526	2.482	1608	1604	9.99±0.27a	9.90±0.08a	-	-	-
B76	[6066-49-5]	3-N-Butylphthalide	48.6499	0.875	2585	2585	10.01±0.04b	10.02±0.04b	10.07±0.03b	10.02±0.05b	10.19±0.02a
B77	[614-99-3]	Ethyl 2-furoate	28.1777	1.359	1616	1618	-	7.87±0.17b	7.31±0.05b	-	11.53±0.84a
B78	[623-70-1]	Ethyl crotonate	10.5856	1.121	1156	1160	-	8.46±0.35a	6.71±0.39b	-	-
B79	[624-13-5]	Propyl octanoate	24.0500	2.522	1511	1510	10.13±0.07b	10.27±0.09b	10.57±0.24b	10.20±0.07b	12.75±0.41a
B80	[624-17-9]	Diethyl azelate	48.2522	1.458	2208	2208	9.89±0.02a	9.94±0.02a	9.92±0.02a	9.90±0.01a	9.94±0.03a
B81	[626-77-7]	Caproic acid propyl ester	16.1163	2.21	1311	1316	13.36±0.01b	13.39±0.02b	13.41±0.01b	13.43±0.02b	13.53±0.06a
B82	[626-82-4]	Butyl hexanoate	17.5858	2.248	1348	1407	10.13±0.05	-	-	-	-
B83	[627-90-7]	Ethyl undecanoate	32.5832	2.514	1736	1739	10.34±0.07a	10.30±0.07a	10.07±0.04b	10.14±0.04b	10.15±0.06b
B84	[628-97-7]	Palmitic Acid Ethyl Ester	49.5165	2.699	2251	2251	30.82±0.06a	26.91±0.19b	22.33±0.73d	23.74±1.67cd	24.43±0.34c
B85	[6290-37-5]	Hexanoic Acid, 2-Phenylethyl Ester	46.9190	1.582	2164	2164	-	10.08±0.11	-	-	-
B86	[6309-51-9]	Isoamyl Laurate	43.7777	3.186	2067	2062	10.18±0.01a	10.22±0.09a	10.13±0.01ab	10.04±0.01b	10.10±0.04ab
B87	[6378-65-0]	Hexanoic Acid hexyl ester	27.7192	2.510	1604	1605	9.84±0.00a	-	9.87±0.07a	9.93±0.06a	9.89±0.06a

B88	[64187-83-3]	Ethyl 3Z-Hexenoate	15.3831	1.737	1292	1292	15.45±0.20c	16.02±0.14b	15.05±0.22c	16.36±0.32b	17.92±0.32a
B89	[659-70-1]	3-Methylbutyl 3-methylbutanoate	15.1857	2.206	1287	1293	9.88±0.05ab	9.83±0.02b	-	9.86±0.01ab	9.90±0.03a
B90	[706-14-9]	γ-Decalactone	46.0445	1.528	2138	2138	-	-	9.88±0.03a	9.91±0.02a	-
B91	[7367-82-0]	Ethyl (E)-2-octenoate	25.4499	2.056	1546	1540	9.96±0.04a	9.95±0.02a	9.94±0.01a	9.92±0.01a	9.98±0.03a
B92	[7367-88-6]	Ethyl trans-2-decenoate	33.2477	2.284	1754	1753	9.90±0.02a	-	9.86±0.03b	9.88±0.00ab	-
B93	[7367-90-0]	Ethyl 3-hydroxyoctanoate	37.9165	1.066	1887	1884	10.40±0.02bc	10.42±0.06bc	10.69±0.15ab	10.35±0.02c	10.91±0.22a
B94	[7452-79-1]	Ethyl 2-methylbutyrate	7.5165	1.388	1045	1052	6.02±0.30abc	5.41±0.26c	6.34±0.13ab	5.65±0.19bc	6.75±0.56a
B95	[76649-16-6]	Ethyl trans-4-decenoate	30.7829	2.149	1686	1683	62.45±1.93b	71.93±1.30a	54.09±0.83c	48.41±0.04d	51.62±0.58c
B96	[93-89-0]	Ethyl benzoate	29.8497	1.405	1661	1658	14.12±0.03a	13.63±0.01c	13.81±0.13b	13.90±0.08b	13.88±0.09b
B97	[96-48-0]	Butyrolactone	28.3817	0.955	1622	1632	32.43±0.74a	26.87±0.15b	24.19±0.34c	27.00±0.78b	19.56±0.75d
B98	[97-62-1]	Ethyl isobutyrate	6.2497	1.029	985	961	18.51±0.14c	12.14±0.12e	15.42±0.49d	24.10±0.53b	25.99±0.06a
B99	[97-64-3]	Ethyl lactate	17.514	0.900	1346	1347	-	-	9.74±1.02a	11.03±1.21a	11.77±2.51a
Subtotal							1596.09±6.43d	1789.68±16.23b	1741.16±21.64c	1521.10±11.26e	1964.86±12.73a
C_6 Compounds											
C1	[111-27-3]	1-Hexanol	17.7189	0.685	1351	1355	1119.07±30.47cd	1265.06±23.42b	1159.88±15.52c	1070.02±10.28d	1904.29±26.49a
C2	[928-94-9]	(Z)-2-Hexen-1-Ol	20.2484	0.837	1415	1415	7.14±0.36d	3.83±0.40e	8.21±0.39c	10.12±0.59b	20.47±0.52a
C3	[928-97-2]	3-Hexen-1-Ol	18.2478	0.910	1365	1367	61.08±0.40d	63.05±0.85d	74.52±0.07c	79.24±2.32b	85.32±0.73a
Subtotal							1183.00±30.19d	1329.64±23.83b	1239.33±15.28c	1150.96±12.64d	2001.89±26.70a
Acids											
D1	[116-53-0]	2-methyl-Butanoic acid	30.1162	0.502	1668	1662	187.98±1.03a	172.49±2.91b	126.08±2.33c	177.78±12.41ab	182.89±1.68ab
D2	[124-07-2]	Octanoic acid	43.5166	0.509	2057	2060	34.23±0.89c	42.42±2.21b	42.58±3.08b	30.11±1.15d	51.00±0.17a
D3	[142-62-1]	Hexanoic acid	36.4481	0.572	1844	1846	131.82±2.68b	164.59±1.95a	-	-	83.92±1.82c
D4	[143-07-7]	Dodecanoic acid	56.1831	0.594	2496	2496	14.29±0.43c	15.55±0.32b	9.93±0.18d	9.00±0.69e	17.68±0.21a
D5	[14436-32-9]	9-Decenoic acid	52.0523	0.380	2345	2345	9.63±0.55b	12.63±0.04a	12.23±0.75a	8.03±0.28c	10.19±0.43b
D6	[334-48-5]	n-Decanoic acid	50.1167	0.538	2276	2276	137.77±2.51d	155.26±3.60b	149.30±1.02c	90.01±0.74e	175.72±3.66a
Subtotal							504.23±4.68a	518.75±8.45a	333.69±5.50c	309.10±12.13d	469.02±3.83b
Aldehydes											
E1	[100-52-7]	Benzaldehyde	24.3140	1.219	1517	1520	13.12±0.19c	13.19±0.47c	14.81±0.34b	12.91±0.25c	20.58±0.06a
E2	[101-86-0]	2-(Phenylmethylene)-octanal	52.4525	1.447	2309	2309	13.54±0.07a	13.47±0.02a	13.46±0.02a	13.47±0.06a	13.54±0.04a
E3	[112-31-2]	Decanal	23.1806	2.163	1489	1498	13.88±0.21b	13.91±0.09b	14.21±0.12a	-	-
E4	[112-54-9]	Dodecanal	31.3833	2.142	1703	1711	13.69±0.10a	13.69±0.03a	13.93±0.27a	13.73±0.00a	13.91±0.16a

E5	[122-78-1]	Benzeneacetaldehyde	28.8481	1.187	1634	1641	11.56±0.15bc	10.83±0.49c	12.20±0.42ab	12.61±0.30a	9.73±0.39d
E6	[124-13-0]	Octanal	15.0444	2.008	1283	1289	5.42±0.02a	-	5.50±0.07a	5.46±0.02a	5.11±0.03b
E7	[124-19-6]	Nonanal	18.9167	1.886	1382	1391	4.07±0.14c	4.43±0.02b	4.43±0.05b	4.52±0.14b	5.19±0.13a
E8	[18829-56-6]	(E)-2-Nonenal	24.8481	1.678	1531	1534	12.54±0.01a	-	-	-	12.58±0.05a
E9	[28785-06-0]	4-Propyl-benzaldehyde	35.6522	1.244	1821	1820	14.01±0.20a	-	13.82±0.12ab	13.65±0.07b	-
E10	[3155-71-3]	2-Methyl-4-(2,6,6-trimethyl-1-cyclohexen-1-yl)-2-Butenal	29.0475	0.211	1639	1639	-	-	-	13.50±0.02	-
E11	[3299-32-9]	2,4,5-Trimethyl-1,3-dioxolane	5.5807	1.109	940	994	188.48±1.86a	176.87±8.79b	-	-	55.01±1.25c
E12	[432-25-7]	-cyclocitral	28.1165	1.875	1615	1611	13.46±0.01b	13.45±0.00b	13.48±0.01b	13.45±0.01b	13.57±0.04a
E13	[4748-78-1]	4-Ethyl-benzaldehyde	31.3164	1.313	1700	1721	16.43±1.25a	14.14±0.08b	16.39±0.59a	12.60±0.25c	-
E14	[57194-69-1]	(Z)-3-Phenylacrylaldehyde	36.9162	1.097	1858	1884	12.89±0.18a	11.42±0.55b	11.47±0.07b	12.74±0.21a	13.48±0.28a
E15	[5973-71-7]	3,4-Dimethylbenzaldehyde	35.0499	1.265	1804	1790	-	18.27±0.77c	28.54±0.64b	18.61±0.31c	47.96±1.42a
E16	[620-23-5]	m-Tolualdehyde	28.9828	1.213	1638	1627	-	-	-	-	7.72±1.89
	Subtotal						155.36±1.25b	174.08±2.33a	129.07±1.05d	102.51±0.48e	134.87±0.37c
	Ketones										
F1	[108-83-8]	2,6-Dimethyl-4-heptanone	10.8523	1.687	1165	1191	14.28±0.25d	13.85±0.22d	16.79±0.32b	15.26±0.33c	17.85±0.14a
F2	[112-12-9]	2-Undecanone	27.3111	2.299	1594	1598	12.07±0.01a	11.63±0.06c	11.38±0.05d	11.79±0.07bc	11.84±0.14b
F3	[1534-26-5]	3-Tridecanone	33.9190	2.149	1773	1755	10.49±0.08a	10.46±0.03ab	-	-	10.37±0.04b
F4	[18787-66-1]	3-Pentadecanone	41.0477	2.508	1981	1955	10.37±0.04a	10.34±0.02a	-	10.37±0.05a	10.34±0.01a
F5	[2345-28-0]	2-Pentadecanone	42.2495	2.226	2018	2019	10.44±0.03ab	10.41±0.02b	10.44±0.03ab	10.42±0.01b	10.51±0.06a
F6	[23696-85-7]	Damascenone	33.4524	1.854	1760	1801	8.30±0.01c	8.44±0.02a	8.36±0.02bc	8.32±0.01c	8.41±0.06ab
F7	[2408-37-9]	2,2,6-Trimethyl-cyclohexanone	15.8497	2.071	1304	1317	-	-	-	-	10.94±0.16
F8	[502-69-2]	Fitone	45.5828	2.442	2131	2131	10.55±0.06a	10.64±0.11a	10.52±0.05a	10.51±0.09a	10.69±0.08a
F9	[6175-49-1]	2-Dodecanone	31.3189	1.932	1701	1698	-	-	10.33±0.01b	10.43±0.04a	10.38±0.03a
F10	[6901-97-9]	Ionone, A	24.7194	2.502	1528	1525	10.35±0.03b	-	10.43±0.05a	10.36±0.02b	10.36±0.01b
F11	[78-59-1]	Isophorone	27.1855	1.305	1590	1591	-	-	-	-	13.19±0.47
F12	[821-55-6]	2-Nonanone	18.8498	1.765	1380	1390	11.09±0.16c	10.97±0.09c	12.52±0.59b	12.18±0.11b	13.21±0.19a
F13	[88-29-9]	Versalide	51.3166	2.389	2325	2325	10.42±0.01a	10.36±0.02a	10.39±0.05a	10.39±0.03a	10.43±0.03a
F14	[937-30-4]	4-Ethylacetophenone	35.7831	1.328	1825	1867	11.00±0.19a	-	-	10.83±0.05a	10.78±0.08a
F15	[96-04-8]	Acetyl valeryl	9.9140	1.304	1135	1152	-	-	14.42±1.76a	13.39±0.76a	-
F16	[110-93-0]	Methylheptenone	16.9816	1.566	1333	1339	2.84±0.01c	2.85±0.02c	3.13±0.03b	2.88±0.06c	3.41±0.02a
	Subtotal						86.46±0.52c	87.48±0.39c	87.19±1.10c	98.16±1.20b	113.63±0.78a

Terpenes												
G1	[106-22-9]	Citronellol	33.6494	1.022	1765	1765	14.73±0.12b	14.83±0.13b	15.38±0.10b	14.13±0.33b	18.24±1.04a
G2	[106-24-1]	Geraniol	36.5166	0.927	1846	1847	13.85±0.47b	14.13±0.21b	14.73±0.65b	11.07±0.24c	17.89±0.21a
G3	[106-25-2]	Nerol	34.8525	0.836	1798	1797	8.68±0.39b	8.85±0.46b	10.12±0.28a	9.60±0.58ab	10.10±0.25a
G4	[141-12-8]	Neryl acetate	33.1858	1.721	1752	1724	-	10.30±0.11	-	-	-
G5	[502-61-4]	*α*-Farnesene	32.8497	2.689	1743	1745	17.17±0.02a	17.20±0.11a	16.97±0.06a	16.99±0.09a	17.03±0.32a
G6	[78-70-6]	Linalool	25.3165	1.100	1543	1547	2.23±0.17c	2.50±0.16c	3.54±0.03b	2.49±0.26c	3.94±0.09a
G7	[98-55-5]	*α*-Terpineol	31.1190	1.062	1695	1697	6.17±0.15a	6.49±0.16a	6.15±0.15a	6.14±0.08a	6.44±0.13a
G8	[99-86-5]	*α*-Terpinene	24.1113	2.884	1513	1480	8.20±0.29a	-	-	8.35±0.15a	-
G9	[99-87-6]	*p*-Cymene	16.0522	1.919	1309	1272	-	2.03±0.03a	-	2.04±0.02a	-
Subtotal							64.46±0.94c	68.52±0.66b	66.89±0.74b	62.14±0.63c	73.64±1.76a
C_{13}-Norisoprenoids											
H1	[23726-93-4]	*β*-Damascenone	35.4498	1.844	1816	1823	12.27±0.20c	13.97±0.17b	13.90±0.25b	12.00±0.10c	15.27±0.05a
H2	[689-67-8]	5,9-Undecadien-2-one, 6,10-dimethyl-	36.6481	1.907	1850	1841	10.59±0.03a	10.48±0.01a	10.52±0.07a	10.49±0.04a	10.56±0.10a
H3	[79-77-6]	trans-*β*-Ionone	39.5166	1.821	1935	1941	5.32±0.03b	5.40±0.03b	5.40±0.13b	5.27±0.01b	5.70±0.12a
Subtotal							28.18±0.23c	29.84±0.18b	29.82±0.38b	27.75±0.07c	31.53±0.26a
Other compounds											
H4	[1222-05-5]	Cyclopenta[G]-2-Benzopyran, 1,3,4,6,7,8-Hexahydro-4,6,6,7,8,8-Hexamethyl-	51.5162	2.399	2326	2326	10.34±0.15ab	10.54±0.41a	10.03±0.16ab	9.91±0.02b	10.06±0.02ab
H6	[36431-72-8]	1-Oxaspiro [4.5] Dec-6-Ene, 2,6,10,10-Tetramethyl-	24.9166	3.036	1533	1526	10.39±0.14a	10.49±0.08a	10.43±0.01a	10.45±0.01a	10.52±0.01a
H8	[41678-29-9]	Edulan I	27.8523	2.386	1608	1611	-	10.32±0.01	-	-	-
H5	[18794-84-8]	(E)-B-Farnesene	29.7829	2.826	1660	1665	18.75±0.15a	18.81±0.12a	17.70±0.25c	18.22±0.11b	17.86±0.09c
H7	[3777-69-3]	2-Pentyl-Furan	12.0476	2.032	1203	1232	10.49±0.15a	10.34±0.12ab	10.16±0.02b	10.26±0.09ab	10.51±0.16a
H9	[464-17-5]	Bicyclo [2.2.1]Hept-2-Ene, 1,7,7-Trimethyl-	24.0525	2.448	1511	1513	7.55±0.11c	8.28±0.10a	8.09±0.09b	-	8.36±0.04a
Subtotal							36.41±0.28e	48.77±0.16b	45.15±0.28c	40.09±0.21d	53.29±0.25a
Total							13377.33±47.32b	12817.27±72.74c	11043.83±38.23d	10687.19±42.07e	13520.80±10.53a

Note: Different letters represent the significant differences between the producing areas ($p < 0.05$).
